# Individuals with problem gambling and obsessive-compulsive disorder learn through distinct reinforcement mechanisms

**DOI:** 10.1371/journal.pbio.3002031

**Published:** 2023-03-14

**Authors:** Shinsuke Suzuki, Xiaoliu Zhang, Amir Dezfouli, Leah Braganza, Ben D. Fulcher, Linden Parkes, Leonardo F. Fontenelle, Ben J. Harrison, Carsten Murawski, Murat Yücel, Chao Suo

**Affiliations:** 1 Centre for Brain, Mind and Markets, The University of Melbourne, Carlton, Australia; 2 Center for the Promotion of Social Data Science Education and Research, Hitotsubashi University, Tokyo, Japan; 3 BrainPark, Turner Institute for Brain and Mental Health, School of Psychological Sciences, and Monash Biomedical Imaging Facility, Monash University, Clayton, Australia; 4 Data61, Commonwealth Scientific and Industrial Research Organisation (CSIRO), Sydney, Australia; 5 School of Physics, The University of Sydney, Sydney, Australia; 6 Department of Bioengineering, School of Engineering & Applied Science, University of Pennsylvania, Philadelphia, Pennsylvania, United States of America; 7 Melbourne Neuropsychiatry Centre, Department of Psychiatry, The University of Melbourne, Carlton, Australia; National Institute on Drug Abuse Intramural Research Program, UNITED STATES

## Abstract

Obsessive-compulsive disorder (OCD) and pathological gambling (PG) are accompanied by deficits in behavioural flexibility. In reinforcement learning, this inflexibility can reflect asymmetric learning from outcomes above and below expectations. In alternative frameworks, it reflects perseveration independent of learning. Here, we examine evidence for asymmetric reward-learning in OCD and PG by leveraging model-based functional magnetic resonance imaging (fMRI). Compared with healthy controls (HC), OCD patients exhibited a lower learning rate for worse-than-expected outcomes, which was associated with the attenuated encoding of negative reward prediction errors in the dorsomedial prefrontal cortex and the dorsal striatum. PG patients showed higher and lower learning rates for better- and worse-than-expected outcomes, respectively, accompanied by higher encoding of positive reward prediction errors in the anterior insula than HC. Perseveration did not differ considerably between the patient groups and HC. These findings elucidate the neural computations of reward-learning that are altered in OCD and PG, providing a potential account of behavioural inflexibility in those mental disorders.

## Abstract 2

A brain imaging study reveals that obsessive-compulsive disorder and problem gambling are associated with distinct patterns of learning from positive and negative reward prediction errors, providing a neurocomputational account of abnormal inflexible behaviors in those disorders.

## Introduction

A central challenge in computational psychiatry is elucidating the fundamental brain processes underlying mental disorders [1–4]. By linking formal models of behaviour with neural data, researchers have started to uncover the basic neural mechanisms of psychiatric symptoms such as anhedonia [5,6] and hallucination [7].

Learning the value of a course of action is critical for appropriate decision-making [8]. The reinforcement learning (RL) account of decision-making posits that such learning is driven by reward prediction errors [9]. The errors are defined as the discrepancy between the actual and expected reward. They are encoded in a network of brain regions, including the midbrain [10,11], striatum [12,13], medial prefrontal cortex (mPFC) [14,15], and anterior insula [16,17] (see [18] for a recent meta-analysis). Apart from clarifying our basic understanding of decision-making, recent studies in computational psychiatry have explored the brain mechanisms related to psychiatric symptoms based on RL frameworks [19–23].

Both obsessive-compulsive disorder (OCD) and pathological gambling (PG) are characterised by deficits in behavioural flexibility [24–30]. For example, a core symptom of OCD is repetitive behaviours (e.g., excessive hand washing and endless rechecking). Individuals with PG experience difficulty in stopping gambling behaviour despite known adverse consequences. These symptoms may be interpreted as a manifestation of the concept of behavioural addiction [31]. Furthermore, our previous study identified common dimensions (i.e., disinhibition, impulsivity, and compulsivity) of various symptoms across the two disorders, which are located on a continuum with healthy participants [32]. On the other hand, the concept of behavioural addiction remains controversial despite the similarities between PG and OCD. Indeed, DSM-5 classifies gambling disorder as an addictive disorder that is separate from OCD and impulse-control disorders [27,30,33,34]. Whether common or distinct neurocomputational processes mediate the behavioural inflexibility in OCD and PG remains elusive.

One influential explanation suggests that behavioural inflexibility can reflect perseveration independent of the outcomes of decisions [35,36]. That is, inflexibility results from a tendency to repeat the same action, regardless of its outcome. In other words, the same action is repeated even though it caused harmful consequences in the past. Yet, previous studies in computational psychiatry have often failed to detect increased perseveration in OCD and PG patients [37–41]. An alternative account posits that inflexibility reflects asymmetric RL, i.e., overlearning from better-than-expected outcomes and underlearning from worse-than-expected outcomes [42,43]. In formal terms, the learning rate—the extent to which new information (e.g., reward, loss, and neutral outcome) modulates future behaviour—is different for value updating from positive and negative reward prediction errors. This asymmetric learning results in excessive reinforcement and deficient devaluation of the current action, leading to inflexibility of choice [43]. However, examinations of the asymmetric RL account in OCD and PG remain sparse.

In this study, we hypothesised that neural computations underlying RL are altered in OCD and PG. There is supporting evidence for this hypothesis. Both OCD and PG are associated with altered processing of reward and loss information in a number of brain regions, including the striatum, mPFC, adjacent anterior cingulate cortex (ACC), and insula [44–48] (but see [49]). Two recent studies have combined a formal RL model with a neuroimaging experiment to demonstrate an abnormal signal of reward prediction error in the ACC [37,50]. Furthermore, the neurotransmitter dopamine, which signals reward prediction errors in the context of RL, has been implicated in the physiopathology of OCD and PG [50–52].

**Fig 1:**
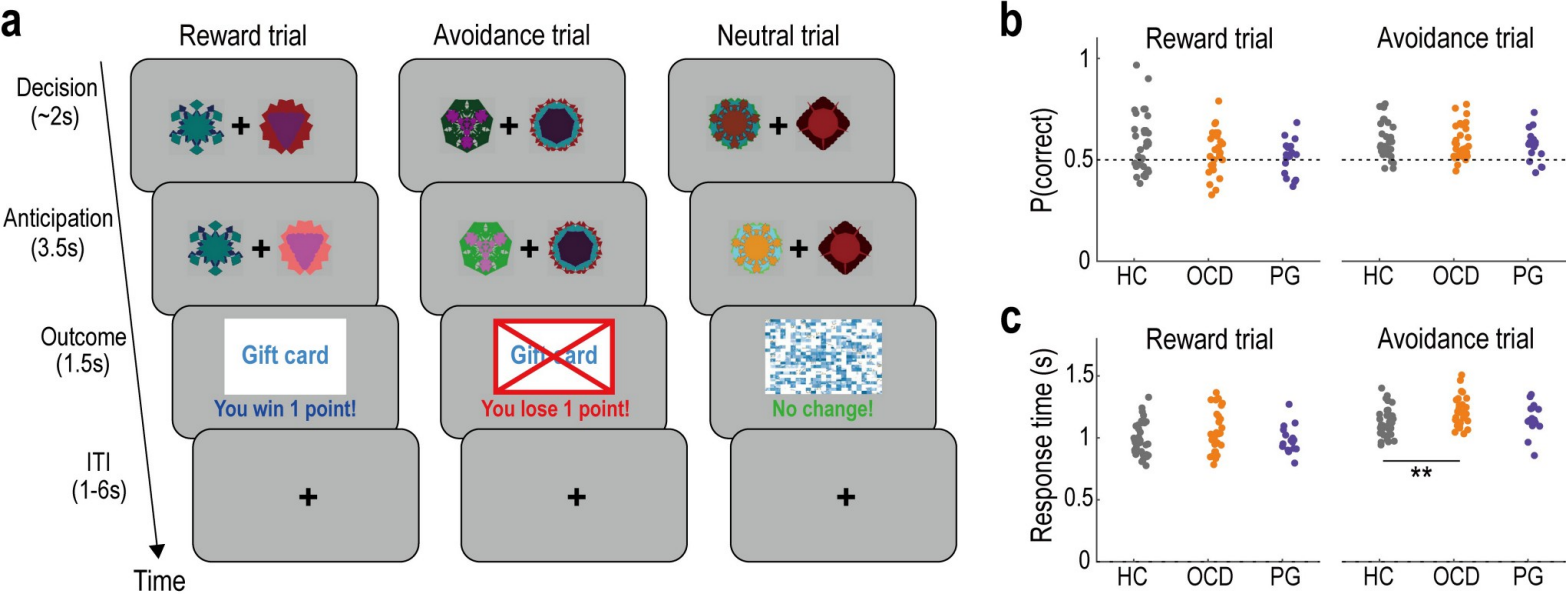
Experimental task and basic behaviour. (a) Illustration of the probabilistic instrumental learning task. In each reward trial, the participants select one of the two options (the fractal images) and either receive a reward (“You win 1 point!”) or nothing, depending on the probability assigned to the chosen option. In each avoidance trial, participants receive either a loss (“You lose 1 point!”) or nothing. In each neutral trial, a visual image not associated with monetary incentives is presented with the message (“No change!”). ITI, intertrial interval. (b) The proportion of correct choices in reward and avoidance trials. Here, the correct choice is defined as selecting the option that provides a higher reward (or lower loss) probability in the given trial. HC, healthy control; OCD, obsessive-compulsive disorder; PG, pathological gambling. (c) Mean response time (seconds) in the reward and avoidance trials. **P < 0.01, two-tailed Welch’s *t* test, Bonferroni-corrected for the two tests performed: HC vs. OCD, and HC vs. PG. Summary data to reproduce the figure are available at https://osf.io/v7em5/.

To test the hypothesis, we recruited 29 patients with OCD, 17 patients with PG, and 34 healthy controls (HCs). The three groups of participants performed a probabilistic instrumental learning task while undergoing MRI (Fig 1A). The experimental task consisted of two sessions, with three conditions (reward, avoidance, and neutral trials) interleaved across trials in a pseudo-randomised order. That is, one of the conditions was presented at random on each trial. Participants repeatedly made choices to earn rewards in the reward trials and to avoid losses in the avoidance trials. On each trial, they selected one of two stimuli, and then received a reward/loss or nothing depending on the probability assigned to the chosen option (Fig 1A). To earn rewards and avoid losses as much as possible, they needed to learn the reward/loss probability of each stimulus through experience. In the neutral trials, a visual image not associated with reward or loss was presented, depending on the participants’ choices. By computational modelling of behaviour, we examined whether behavioural effects of reward prediction errors (i.e., learning rates in RL) are altered in OCD and PG. Furthermore, we sought to identify neural underpinnings of RL anomalies, focusing on prediction error signals in the striatum, mPFC, ACC, and insula.

## Results

### Demographics and basic behaviour

There were no significant differences between the OCD/PG and HC groups in age (Welch’s *t* test; HC versus OCD: *t* = 0.900 and *P*_*corr*_ = 0.744; HC versus PG: *t* = 0.274 and *P*_*corr*_ = 1.000), sex ratio (Fisher’s exact test; HC versus OCD: *P*_*corr*_ = 1.000; HC versus PG: *P*_*corr*_ = 1.000), and IQ (Welch’s *t* test; HC versus OCD: *t* = 0.456 and *P*_*corr*_ = 1.000; HC versus PG: *t* = 0.522 and *P*_*corr*_ = 1.000). As expected, the questionnaire-based OCD symptoms (i.e., the score on the Obsessive-Compulsive Inventory-Revised scale [53]) were more severe in the OCD group than in the HC and PG groups (Welch’s *t* test; OCD versus HC: *t* = 11.096 and *P*_*corr*_ < 0.001; OCD versus PG: *t* = 5.266 and *P*_*corr*_ < 0.001). Moreover, the PG group exhibited problematic gambling (i.e., the score on the Problem Gambling Severity Index [54]) to a higher extent than the HC and OCD groups (Welch’s *t* test; PG versus HC: *t* = 7.853 and *P*_*corr*_ < 0.001; PG versus OCD: *t* = 7.677 and *P*_*corr*_ < 0.001). Additional details are provided in S1 Table and S1 Fig.

Overall task performance did not differ between the OCD/PG and HC groups (Fig 1B). There were no significant differences in the proportion of correct choices (i.e., selection of the option with the higher/lower reward/loss probability) in the reward trials (Welch’s *t* test; HC versus OCD: *t* = 1.553 and *P*_*corr*_ = 0.251; HC versus PG: *t* = 2.197 and *P*_*corr*_ = 0.066) or the avoidance trials (Welch’s *t* test; HC versus OCD: *t* = 0.508 and *P*_*corr*_ = 1.000; HC versus PG: *t* = 1.065 and *P*_*corr*_ = 0.589), while the proportion in the reward trials was significantly lower in the PG group than in the HC group without the multiple comparison correction (*P* = 0.033). Furthermore, we observed no significant differences in response time between the OCD/PG and HC groups, except that OCD patients had longer reaction time than HC in the avoidance trials (Fig 1C; Welch’s *t* test; reward trial: HC versus OCD, *t* = 1.484 and *P*_*corr*_ = 0.288; HC versus PG, *t* = 0.223 and *P*_*corr*_ = 1.000; and avoidance trial: HC versus OCD, *t* = 2.954 and *P*_*corr*_ = 0.009; HC versus PG, *t* = 0.840 and *P*_*corr*_ = 0.815).

### Behaviour: The effect of the past outcome and choice

**Fig 2:**
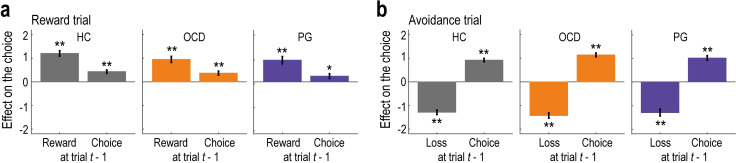
Model-neutral regression analysis on the behaviour. (a) Effects of past rewards and choices on current behaviour in reward trials (mean ± SEM, estimated using the three generalised linear mixed-effect models, GLMM1). **P < 0.01 and *P < 0.05, two-tailed Welch’s *t* test, Bonferroni-corrected for the two tests performed in each model. HC, healthy control; OCD, obsessive-compulsive disorder; PG, pathological gambling. (b) Effects of past losses and choices on current behaviour in avoidance trials. The format is the same as in (a). Summary data to reproduce the figure are available at https://osf.io/v7em5/.

We next analysed the trial-by-trial choice data to confirm that participants’ behaviour was driven by past reward and loss outcomes. Furthermore, we also examined how past choices affected their behaviour [36]. Generalised linear mixed models (GLMM1) revealed that the past reward and loss outcomes had significant impacts on current choice behaviour (Fig 2). Consistent with the RL framework, past rewards in the reward trials had a significantly positive effect (Fig 2A; HC: *b* = 1.204, *t* = 10.454, and *P*_*corr*_ < 0.001; OCD: *b* = 0.952, *t* = 7.409, and *P*_*corr*_ < 0.001; PG: *b* = 0.920, *t* = 5.977, and *P*_*corr*_ < 0.001), and past losses in the avoidance trials had a significantly negative effect (Fig 2B; HC: *b* = −1.290, *t* = 13.150, and *P*_*corr*_ < 0.001; OCD: *b* = −1.428, *t* = 12.832, and *P*_*corr*_ < 0.001; PG: *b* = −1.302, *t* = 9.002, and *P*_*corr*_ < 0.001) across all groups. Past outcomes in the neutral trials had no effect (S2A Fig; HC: *b* = −0.031, *t* = 0.290, and *P*_*corr*_ = 1.000; OCD: *b* = 0.010, *t* = 0.081, and *P*_*corr*_ = 1.000. PG: *b* = −0.119, *t* = 0.756, and *P*_*corr*_ = 0.900), as expected given that they were not associated with any monetary incentives. Furthermore, in both the reward and avoidance trials, the effect of past choices was significantly positive (Fig 2; reward trial: HC, *b* = 0.434, *t* = 5.884, and *P*_*corr*_ < 0.001; OCD, *b* = 0.371, *t* = 4.324, and *P*_*corr*_ < 0.001; PG, *b* = 0.243, *t* = 2.346, and *P*_*corr*_ = 0.038; and avoidance trial: HC, *b* = 0.922, *t* = 13.211, and *P*_*corr*_ < 0.001; OCD, *b* = 1.142, *t* = 13.929, and *P*_*corr*_ < 0.001; PG, *b* = 1.010, *t* = 10.023, and *P*_*corr*_ < 0.001), indicating a tendency to repeat the same choice.

We also tested for group differences (HC versus OCD and HC versus PG) in the effects of past outcomes and choices on behaviour (GLMM2). The additional analyses revealed no differences between the groups in either the reward trials (HC versus OCD: *t* = 1.559 and *P*_*corr*_ = 0.238 for the effect of past outcomes, and *t* = 0.245 and *P*_*corr*_ = 1.000 for the effect of past choices; HC versus PG: *t* = 1.532 and *P*_*corr*_ = 0.251 for the effect of past outcomes, and *t* = 1.360 and *P*_*corr*_ = 0.348 for the effect of past choices) or the avoidance trials (HC versus OCD: *t* = 0.913 and *P*_*corr*_ = 0.723 for the effect of past outcomes, and *t* = 2.093 and *P*_*corr*_ = 0.073 for the effect of past choices; HC versus PG: *t* = 0.002 and *P*_*corr*_ = 1.000 for the effect of past outcomes, and *t* = 0.847 and *P*_*corr*_ = 0.794 for the effect of past choices) (S2B and S2C Fig).

These results were maintained when different lengths of the outcome and choice history were considered in the GLMMs. For example, when the previous three trials were considered, the total effects of past rewards/losses (i.e., the sum of the regression coefficients over the past three trials: *b*_*sum*_) were significantly positive/negative (reward trial: HC, *b*_*sum*_ = 1.392, *F* = 49.479, and *P*_*corr*_ < 0.001; OCD, *b*_*sum*_ = 1.612, *F* = 53.293, and *P*_*corr*_ < 0.001; PG, *b*_*sum*_ = 1.284, *F* = 25.782, and *P*_*corr*_ < 0.001; and avoidance trial: HC, *b*_*sum*_ = −1.964, *F* = 131.317, and *P*_*corr*_ < 0.001; OCD, *b*_*sum*_ = −1.998, *F* = 105.714, and *P*_*corr*_ < 0.001; PG, *b*_*sum*_ = −2.114, *F* = 63.258, and *P*_*corr*_ < 0.001). Furthermore, no significant differences were found between the groups in either the reward trials (HC versus OCD: *F* = 0.245 and *P*_*corr*_ = 1.000 for the effect of past outcomes, and *F* = 0.068 and *P*_*corr*_ = 1.000 for the effect of past choices; HC versus PG: *F* = 0.259 and *P*_*corr*_ = 1.000 for the effect of past outcomes, and *F* = 4.155 and *P*_*corr*_ = 0.084 for the effect of past choices) or the avoidance trials (HC versus OCD: *F* = 0.007 and *P*_*corr*_ = 1.000 for the effect of past outcomes, and *F* = 1.383 and *P*_*corr*_ = 0.480 for the effect of past choices; HC versus PG: *F* = 0.160 and *P*_*corr*_ = 1.000 for the effect of past outcomes, and *F* = 0.424 and *P*_*corr*_ = 1.000 for the effect of past choices).

### Behaviour: Computational model fits

In theory, the above results can reflect non-trivial interactions among multiple components, such as value learning, perseveration, and stochastic noise in the decision-making process [42]. To disentangle these components, we fitted RL models to participants’ choice data and analysed the best-fitted model (see Methods for details).

RL1 was a conventional RL model, in which the value of the chosen option was updated in proportion to the reward prediction error with learning rate *α*. The learning rate governed the extent to which new reward/loss information was incorporated into the value updating process. RL2 had different learning rates, *α*_(+)_ and *α*_(-)_, for positive and negative reward prediction errors, respectively. RL3 included the perseveration effect, in which the constant bonus, *γ*—denoting the degree of perseveration (i.e., the tendency to repeat the same choice)—was added to the option chosen in the previous trial. RL4 included both differential learning rates and perseveration. In the remainder of this section, we only present RL1, RL2, and RL3, as model recovery analysis on simulated data revealed that the complicated model (RL4) was not well dissociable from the simpler one (RL3) (see S1 Text for detail). For example, RL4 generated the simulated data; however, RL3 and RL4 were identified as the best-fit models with probabilities of 0.37 and 0.47, respectively (S4A Fig), demonstrating poor identifiability between the two models. We confirmed that the key findings (i.e., group differences in the learning rates) did not change whether RL4 is included or not (S1 Text and S4C and S4D Fig). In the model fitting, a hierarchical modelling approach was employed to reduce the estimation noise [55]. Each model’s goodness of fit was assessed using the widely applicable Akaike information criterion (WAIC) [56]. We validated the procedure using parameter- and model-recovery analyses of the simulated data [57] (S3 Fig). We also confirmed that the best-fitted models could replicate the behavioural results obtained in the model-neutral regression analyses (S5 Fig; cf. Figs 1B and 2).

**Fig 3:**
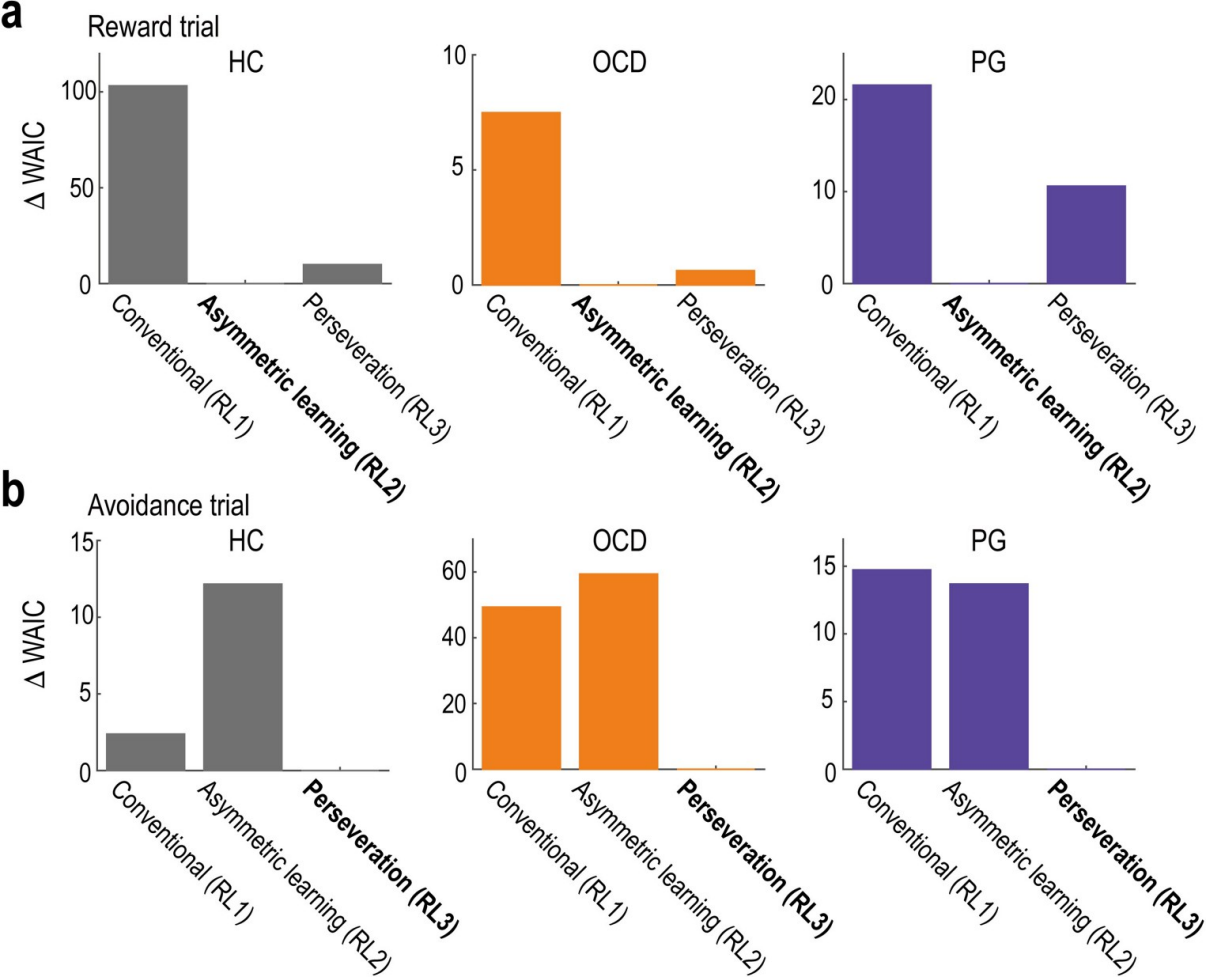
Computational model fitting to behaviour. (a) Model comparison in reward trials. We plotted each model’s WAIC value relative to the best model (note: a smaller value indicates a better fit). The best-fit model is highlighted in bold. HC, healthy control; OCD, obsessive-compulsive disorder; PG, pathological gambling; RL, reinforcement learning; WAIC, widely applicable Akaike information criterion. (b) Model comparison in the avoidance trials. The format is the same as in (a). Summary data to reproduce the figure are available at https://osf.io/v7em5/.

The model comparison revealed that in the reward trials, RL with asymmetric learning rates (RL2) provided the best fit for all three groups (HC, OCD, and PG; Fig 3A). This implies that in reward-seeking decision-making, participants recruited different systems for learning from outcomes that were better (positive prediction error) and worse (negative prediction error) than expected. In contrast, in the avoidance trials, RL with perseveration (RL3) provided the best fit for all groups (Fig 3B). This suggests that in loss-avoidance decision-making, participants employed a common unitary system for learning from positive and negative prediction errors and that they preferred the option that had been previously chosen regardless of its outcome.

In addition to the main RL models, we tested parsimonious models that share a common set of parameters across the two trial types. We also examined a model with motor-perseveration: i.e., the tendency to choose options presented on the same side (left or right) in consecutive trials irrespective of the trial types. Furthermore, we tested another model that involved a context-dependent adaptation of the outcome values [58,59], as well as models that endogenously modulated the learning rate [60,61]. These additional models, however, did not outperform the best fit RL models (see S1 Text for details).

### Behaviour: Parameters of the best-fitted models

**Fig 4:**
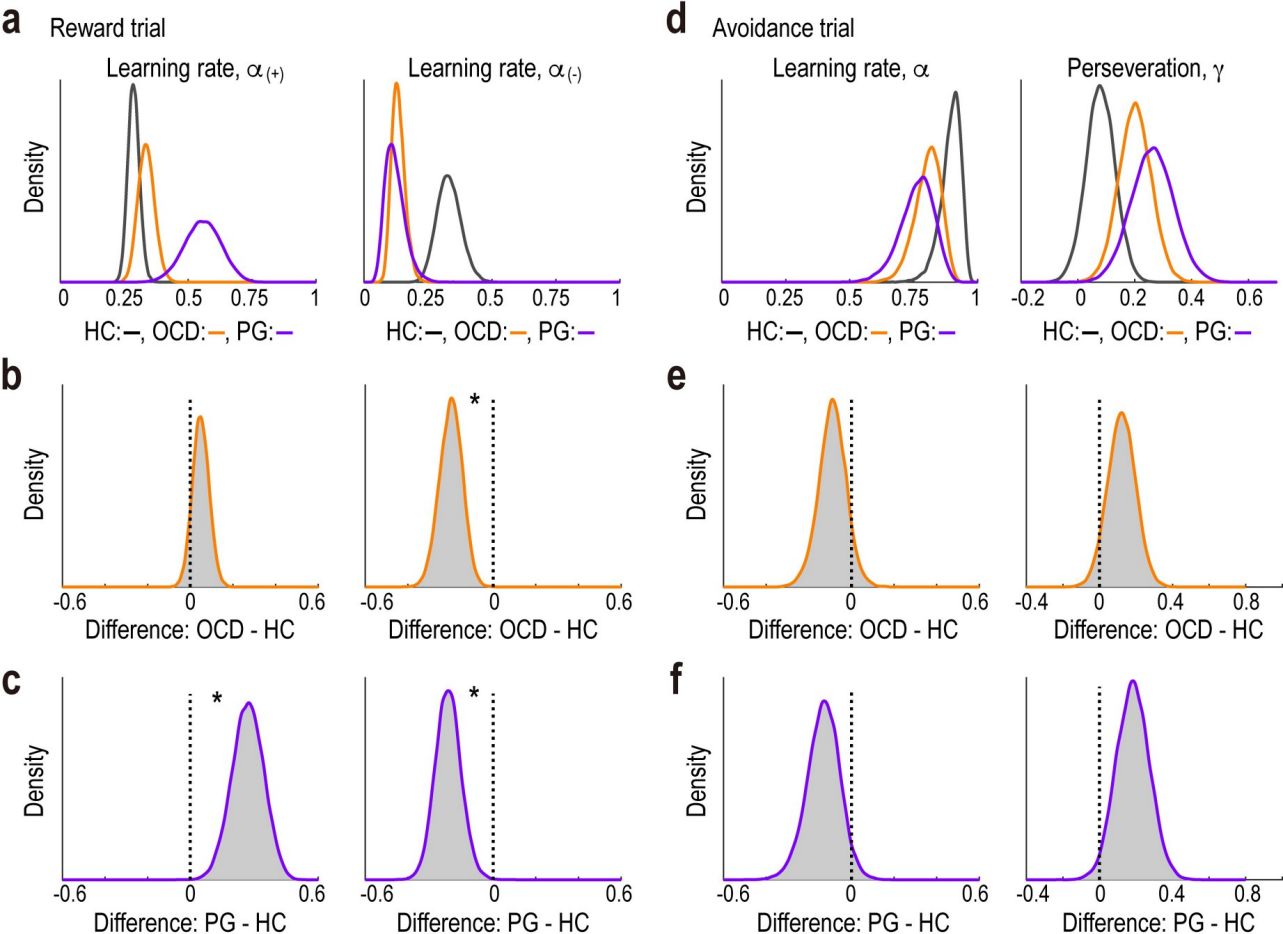
Parameter estimates in the best-fitted models. (a) Posterior distributions of group-level mean parameters of RL2 in reward trials for each group. *Left*, learning rate from the positive reward prediction error, *α*_(+)_. *Right*, learning rate from the negative reward prediction error, *α*_(-)_. *Grey*, healthy control (HC); *orange*, obsessive-compulsive disorder (OCD); *purple*, pathological gambling (PG). (b) Posterior distributions of the difference between the OCD and HC groups in reward trials. *Left*, *α*_(+)_; and *right*, *α*_(-)_. *BF > 20 denotes “very strong” or “strong” evidence [100] for the difference. BF, Bayes factor. (c) Posterior distributions of the difference between the PG and HC groups in reward trials. The format is the same as in (b). (d) Posterior distributions of group-level mean parameters of RL3 in avoidance trials for each group. *Left*: learning rate, *α*; *right*: perseveration, *γ*. The format is the same as in (a). (e) Posterior distributions of the difference between the OCD and HC groups in avoidance trials. *Left*, *α*; *right*, *γ*. The format is the same as in (b). (f) Posterior distributions of the difference between the PG and HC groups in avoidance trials. The format is the same as in (b). Summary data to reproduce the figure are available at https://osf.io/v7em5/.

We further examined whether and how the key decision parameters in the best-fitted models differed between the OCD/PG and HC groups. In the reward trials, compared with the HC group, participants with PG exhibited a higher learning rate for positive reward prediction errors, *α*_(+)_, and a lower rate for negative prediction errors, *α*_(-)_ (Fig 4A and 4C; *α*_(+)_, Bayes factor (BF) = 167.71 and 95% highest density interval (HDI) = [0.12, 0.41]; *α*_(-)_, BF = 40.41 and 95% HDI = [−0.33, −0.09]). Compared to the HC group, we found a lower learning rate for negative prediction errors in the OCD group (Fig 4A and 4B; *α*_(-)_, BF = 104.90 and 95% HDI = [−0.31, −0.09]). In contrast, in the avoidance trials, there was no evidence for differences in key parameter values—learning rate (*α*) and perseveration (*γ*)—between the OCD/PG and HC groups (Fig 4D–4F; HC versus OCD: *α*, BF = 0.47 and 95% HDI = [−0.22, 0.04] and *γ*, BF = 0.06 and 95% HDI = [−0.03, 0.27]; and HC versus PG: *α*, BF = 0.97 and 95% HDI = [−0.29, 0.02] and *γ*, BF = 0.17 and 95% HDI = [0.01, 0.36]). Taken together, these results suggest that PG is characterised by excessive sensitivity (insensitivity) to positive (negative) prediction errors, respectively, whereas OCD is characterised by insensitivity to negative prediction errors in reward-seeking decision-making.

As a robustness check, we obtained the parameter estimates without assuming a hierarchical structure (i.e., each participant’s individual-level parameters were drawn from group-specific higher-level distributions: see S3A Fig). First, assuming all the participants in each group share the same set of the parameter values (S6A Fig), we fitted the best-fitted models by pooling all the participants’ data in each group. Next, assuming each participant has his/her own (independent) set of parameters (S6B Fig), we fitted the models to each participant’s data separately. These additional analyses showed results consistent with those in the original hierarchical model fitting (S6C–S6F Fig): i.e., higher/lower learning rates for positive/negative reward prediction errors in the PG group, and a lower rate for the negative prediction error in the OCD group.

We also examined the associations between questionnaire-based symptom severity and the decision parameters (*α*_(+)_, *α*_(-)_, and *γ*) within each of the three groups. However, we did not find any significant correlations for either reward or avoidance trials (S2 Table), suggesting that the decision parameters were not direct predictors of symptom severity.

### Neuroimaging: Reward prediction error in the reward trials

Before proceeding with the main analyses of interest, we replicated previous well-established findings [62,63]. That is, in healthy people, reward prediction error and expected value signals are represented in the ventral striatum and mPFC. We fitted the RL models to data from the HC group to the (pooled) reward and avoidance trials and confirmed that the ventral striatum encodes the reward prediction error at the time of outcome delivery (S7A–S7C Fig; *P* < 0.05, cluster-level corrected). We also confirmed that the mPFC encodes the value of the chosen option at the time of decision-making (S7D–S7F Fig.; *P* < 0.05, cluster-level corrected).

In the main analysis, we employed a two-step approach. First, we identified the brain regions tracking the reward prediction error independent of diagnosis by averaging the data of all participants. Specifically, we obtained a weighted average, with weights equal to the inverse of the number of participants in each group (see Methods for details), which indicated the regions of interest (ROIs) that were statistically independent of the subsequent across-group comparisons. Next, we tested whether these neural representations differed between the OCD/PG and HC groups.

**Fig 5:**
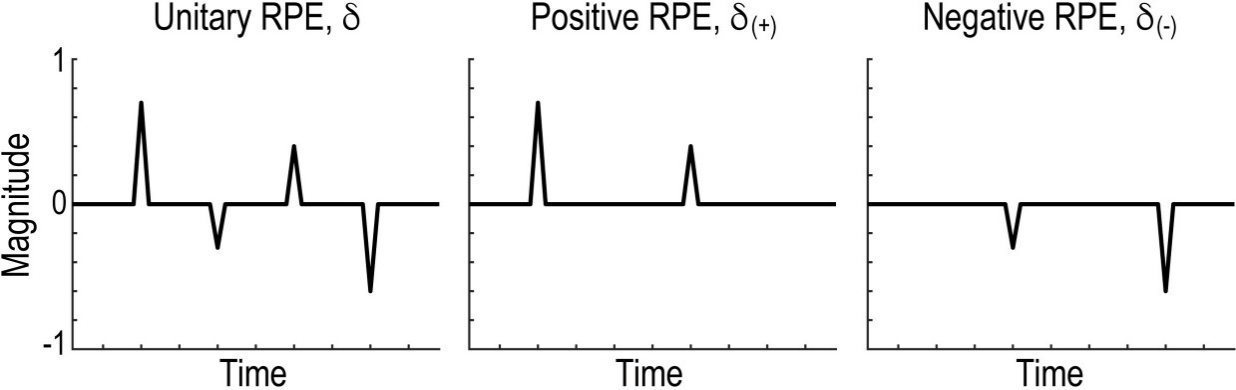
Schematic illustration of the three types of reward prediction error. *Left*, conventional unitary reward prediction error *δ*. *Centre*, positive reward prediction error, *δ*_(+)_ = *max*(*δ*, 0). *Right*, negative reward prediction error, *δ*_(−)_ = *min*(*δ*, 0). RPE, reward prediction error.

The results of the behavioural modelling suggest that in reward-seeking decision-making, learning from positive and negative reward prediction errors was governed by distinct systems (see Fig 5 and the legend for a schematic illustration and formal definition of positive and negative reward prediction errors). Therefore, we hypothesised that the positive and negative parts of the prediction error were processed separately in the brain in the reward trials.

**Fig 6:**
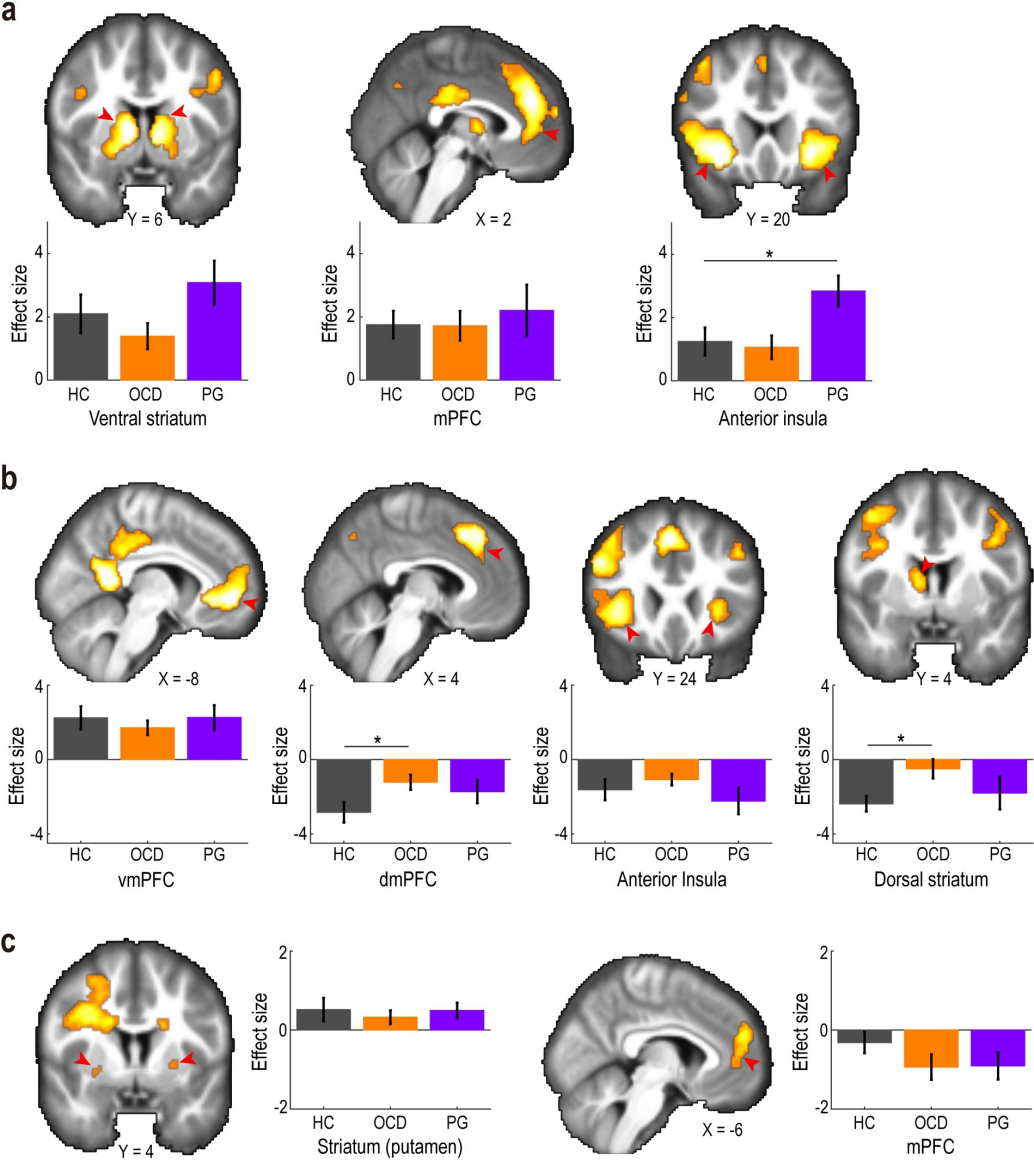
Neural correlates of reward prediction errors. (a) Positive reward prediction error in the reward trials. The activation maps are thresholded at *P* < 0.0005 (uncorrected) for display purposes. Red arrows indicate regions of interest. Bar-plots show effect sizes of the positive reward prediction error in the HC, OCD, and PG groups (mean ± SEM). *Grey*, healthy control (HC); *orange*, obsessive-compulsive disorder (OCD); *purple*, pathological gambling (PG). *P < 0.05 in a two-tailed Welch’s *t* test, Bonferroni-corrected for the two tests performed. mPFC, medial prefrontal cortex. (b) Negative prediction error in the reward trials. vmPFC, ventromedial prefrontal cortex; dmPFC, dorsomedial prefrontal cortex. The format is the same as in (a). (c) Unitary reward prediction error in the avoidance trials. The format is the same as in (a). Summary data to reproduce the figure are available at https://osf.io/v7em5/.

Across the three groups in the reward trials, positive reward prediction errors were significantly correlated with the BOLD signal in the ventral striatum (ventral part of the caudate and putamen extending to the nucleus accumbens), mPFC (Brodmann area, BA 8/32/10), and insula at the time of outcome presentation (Fig 6A; *P* < 0.05, cluster-level corrected; see S3 Table for other activated areas). Comparisons between the HC and PG groups further demonstrated that the neural encoding of positive prediction error in the insula, but not that in the ventral striatum or mPFC, was greater in PG than in HC (Fig 6A and S8 Fig.; insula: *t* = 2.430 and *P*_*corr*_ = 0.039; ventral striatum: *t* = 1.076 and *P*_*corr*_ = 0.577; and mPFC: *t* = 0.491 and *P*_*corr*_ = 1.000). However, no significant difference was detected between the OCD and HC groups in any ROIs (Fig 6A; insula: *t* = 0.324 and *P*_*corr*_ = 1.000; ventral striatum: *t* = 0.963 and *P*_*corr*_ = 0.680; and mPFC: *t* = 0.055 and *P*_*corr*_ = 1.000). We also performed a whole-brain search to explore brain regions that differentially encoded positive prediction errors between the OCD/PG and HC participants. The whole-brain analysis found no regions under the statistical threshold (*P* < 0.05, cluster-level corrected).

Negative reward prediction errors in the reward trials were positively correlated with the BOLD signal in the ventromedial prefrontal cortex (vmPFC, BA 32/10) at the time of outcome presentation across the three groups (Fig 6B; *P* < 0.05, cluster-level corrected). Moreover, the negative prediction error was negatively correlated with the BOLD signal in the dorsomedial prefrontal cortex (dmPFC, BA 8), insula, and dorsal striatum (caudate) (Fig 6B, *P* < 0.05, cluster-level corrected) across the three groups (see S4 Table for a comprehensive list of the activated areas). A comparison of the HC and OCD groups revealed that the encoding of negative prediction errors in the dmPFC and the dorsal striatum was weaker in the OCD group (Fig 6B; dmPFC: *t* = 2.359 and *P*_*corr*_ = 0.043; and dorsal striatum: *t* = 2.831 and *P*_*corr*_ = 0.013; vmPFC: *t* = 0.730 and *P*_*corr*_ = 0.938; and insula: *t* = 0.851 and *P*_*corr*_ = 0.798). The comparison between HC and PG did not reveal significant differences in any of the four ROIs (dmPFC: *t* = 1.330 and *P*_*corr*_ = 0.383; and dorsal striatum: *t* = 0.592 and *P*_*corr*_ = 1.000; vmPFC: *t* = 0.021 and *P*_*corr*_ = 1.000; and insula: *t* = 0.674 and *P*_*corr*_ = 1.000). The whole-brain search also did not detect differential encoding of negative reward prediction errors.

### Neuroimaging: Reward prediction error in the avoidance trials

Since RL with a unitary learning rate (RL3) best explained participants’ behaviour in loss-avoidance decision-making (Fig 3B), we next aimed to identify brain regions encoding the unitary reward prediction error (Fig 5) in avoidance trials. The prediction error was found to be significantly correlated with BOLD signals in the striatum (putamen) and mPFC (BA 10/9) at the time of outcome in the avoidance trials across the three groups (Fig 6C; *P* < 0.05, cluster-level corrected; see S5 Table for other activated areas). The strength of the neural encodings in the striatum and mPFC did not differ significantly between the OCD/PG and HC groups (Fig 6C; striatum: HC versus OCD, *t* = 0.563 and *P*_*corr*_ = 1.000, and HC versus PG, *t* = 0.058 and *P*_*corr*_ = 1.000; and mPFC: HC versus OCD, *t* = 1.478 and *P*_*corr*_ = 0.290, and HC versus PG, *t* = 1.351 and *P*_*corr*_ = 0.371). The whole-brain search revealed no brain regions that differentially encoded reward prediction errors between OCD/PG and HC.

### Neuroimaging: Post hoc analyses

In this experiment, approximately half of the OCD patients were medicated with selective serotonin reuptake inhibitors (SSRIs). We examined how the medication affects key findings in OCD, i.e., attenuated encoding of negative reward prediction errors in the dmPFC and striatum during reward trials (Fig 6B). The additional analysis revealed no significant differences in the encoding strengths of negative prediction errors between medicated and unmedicated OCD patients (dmPFC: *t* = 0.359 and *P* = 0.722; dorsal striatum: *t* = 1.062 and *P* = 0.299), while encoding strengths in medicated OCD patients were between those in unmedicated OCD patients and the HC group (S9 Fig).

A recent perspective [64] by Lebreton and colleagues alerted us to a pitfall in interpreting individual differences in the strength of neural encoding of a computational variable (e.g., reward prediction error): The precise interpretation depends on whether the range of neural activity is adapted to the domain of the variable of interest (i.e., “range-adaptation” coding) or not (i.e., “proportional” coding). The authors claim that typical studies in computational psychiatry, such as our current study, have implicitly assumed proportional coding (i.e., native variables, rather than the standardised variables, are fed into the fMRI analysis), although the coding principle has been unexplored. In other words, the findings of those studies could be spurious if the brain employs a range-adaptation coding. Following the recommendation provided in the perspective [64], we presented the neuroimaging results with z-normalisation of the prediction errors alongside the original ones without normalisation (S10 Fig). The additional data analysis revealed that the effect sizes decreased by an average of 30% from the original ones, while the overall patterns remained unchanged. These findings caution against evaluating the original results of altered neural encodings of reward prediction errors in OCD/PG.

## Discussion

Using an RL framework, this study both identified the existence of RL anomalies in, and elucidated basic neural computations that are altered in patients with OCD and PG. Compared with healthy controls, patients with OCD learned less from worse-than-expected experiences in reward-seeking decision-making, with attenuated representations of the negative reward prediction error in the dmPFC and dorsal striatum. Furthermore, patients with PG exhibited excessive (deficient) learning from better-than- (worse-than-) expected experiences, respectively, reflecting the enhanced representation of positive reward prediction error in the insula. On the other hand, we did not find any behavioural or neural signatures of OCD and PG in loss-avoidance decision-making.

Although behavioural inflexibility is thought to be a hallmark of OCD, the underlying computational mechanisms remain elusive [24,65] (but see [66]). Several studies have addressed this issue by incorporating a so-called perseveration or stickiness parameter, which controls for the tendency to repeat the same choice (regardless of the outcome) in the RL model [37,38]. However, neither of the previous studies nor the present one found any evidence for an increased perseveration parameter in patients with OCD (compared with healthy controls). Instead, a decreased perseveration has been reported in OCD [37,38,40]. In theory, inflexibility can also be captured by asymmetric learning for positive and negative reward prediction errors. That is, excessive learning from better-than-expected experiences and/or deficient learning from worse-than-expected experiences may result in the repetitive choice of the same option [42,43]. Consistent with the theoretical consideration, we demonstrated that, in reward-seeking decision-making, the learning rate for negative reward prediction errors was lower in patients with OCD than in healthy controls. This finding supports the notion of asymmetric learning, and may suggest a novel computational mechanism of behavioural inflexibility in OCD.

Past studies to date have demonstrated altered reward-learning in OCD [38,40,50,67], but the specific pattern of results has been mixed. Marzuki and colleagues [40] demonstrated that adolescents with OCD have lower and higher learning rates from negative and positive reward prediction errors, respectively. The decreased learning rate for negative prediction errors was consistent with our findings, but this study did not observe the increased rate for positive error. On the other hand, another study [38] by Kanen and colleagues reported the opposite pattern: an increased learning rate from negative prediction errors in OCD, contrasting our findings but consistent with the enhanced performance monitoring hypothesis that OCD is associated with increased sensitivity to negative feedback [24,68]. Furthermore, using a novel predictive inference task, Vaghi and colleagues [67] demonstrated an increased unitary learning rate that does not discriminate between positive or negative prediction errors in OCD.

We did not find evidence for altered perseveration in OCD, although previous studies reported a decreased perseveration [37,38,40]. This discrepancy may be due to the differences in tasks. In our task, three pairs of fractal images (corresponding to the three types of trials; see Fig 1A) were interleaved across trials in each session. In this setting, perseveration does not necessarily imply choosing the same option (image) in consecutive trials. In contrast, only one pair of images was presented within a session of the tasks used in the previous studies [37,38,40], where perseveration indicates choices of the same option in consecutive trials. This difference may reconcile the seemingly inconsistent findings. Furthermore, in the present study, we excluded participants who chose only one of the options in some sessions (i.e., never chose the other option), which could affect the null finding of perseveration in OCD.

We found that, in reward-seeking decision-making, the decreased learning rate from worse-than-expected outcomes in OCD was associated with altered neural encoding of negative reward prediction errors in both the dorsal striatum (caudate) and the dmPFC (including the ACC). Specifically, neural activity in those regions was negatively coupled with negative prediction error, and the couplings were weakened in OCD (Fig 6B). The dmPFC finding is broadly consistent with that of a previous study [37] by Hauser and colleagues. They showed an altered negative coupling between ACC activity and reward prediction errors in patients with OCD. The study found that neural activity in the ACC was negatively correlated with unitary reward prediction error independent of diagnosis. The negative correlation was attenuated in patients with OCD compared to healthy controls (i.e., the correlation was highly negative in the healthy controls but not in OCD patients). The pattern of the altered prediction error signals in ACC is partly consistent with what was observed in the dmPFC’s negative prediction error signal (i.e., negative component of the unitary prediction error; see Fig 5). A subsequent study further demonstrated that the neural response to reward prediction errors in the ACC was modulated by a dopamine D2/3 receptor agonist, pramipexole, and a dopamine D2/3 receptor antagonist, amisulpride [50]. The caudate nucleus is a major projecting locus of dopamine neurons in the substantia nigra pars compacta [69] and a core region of the OCD-related neural network [70,71]. For example, reduced resting-state functional connectivity between the caudate and lateral prefrontal cortex is associated with the impairment of cognitive flexibility in patients with OCD [72]. Elevated activity in the caudate has also been implicated in the formation of excessive habits in patients with OCD [73]. Our neuroimaging results, together with the previous findings, suggest that the altered dopaminergic neural responses to reward prediction error in the dmPFC and striatum (caudate) underlie the deficient learning from worse-than-expected experiences in OCD.

Behavioural inflexibility in OCD has been assessed using various tasks [24] including probabilistic reversal learning [37,40], delayed feedback [74], and deterministic set-shifting tasks (e.g., Wisconsin Card Sorting task) [75]. Notably, Gillan and colleagues examined behavioural inflexibility in OCD concerning an imbalance between goal-directed and habitual behaviours [76,77] using tasks that involve two-stage decision-making, devaluation, or contingency degradation [78]. One caveat in this study is whether and how much our findings in the probabilistic instrumental learning tasks can be generalised to other contexts.

The computational modelling of behaviour in patients with PG revealed increased/decreased learning rates for positive/negative reward prediction errors for reward-seeking decision-making. In other words, PG patients learned more from better-than-expected experiences and less from worse-than-expected experiences than healthy controls, which may lead to addictive gambling despite the adverse consequences. The learning-based account has been indirectly suggested by previous studies on neural responses to monetary reward and loss in PG patients [46]. However, no study to date has demonstrated direct evidence. For example, previous studies of RL in PG did not explore asymmetric learning [39,41,79,80], while they employed reward-learning tasks. To our knowledge, the present study is the first to provide direct evidence for this account by fitting a formal RL model with asymmetric learning rates to behaviour.

We found increased insula neural activity in response to positive reward prediction error in PG patients in reward-seeking decision-making. This result is consistent with previous findings that a brain network including the insula is associated with behaviours implicated in PG [81–83]. In PG patients, a craving for gambling is correlated with enhanced reactivity to the gambling cue (but not food cues) in the insula [83]. Furthermore, patients with damage to the insula exhibit less gambling-related cognitive distortions [82]. These findings implicate the insula in PG-related overlearning from positive information and may contribute to the development of novel treatments for PG that target neural activity in the insula.

We did not find behavioural or neural differences between OCD/PG patients and healthy controls in loss-avoidance decision-making. The null results could be due to the small effect size relative to the sample size. We used monetary loss as an aversive stimulus in the experiment, whereas previous studies used electrical shocks [73,84]. The difference in the psychological/neural intensity between the primary (electrical shocks) and secondary (monetary losses) punishments may cause a smaller effect size in our study. More research is needed to further examine avoidance decision-making in OCD and PG.

In this study, participants with OCD and PG performed the same decision-making task in the same environment. This experimental design enabled the elucidation of similarities and differences between OCD and PG. Despite the similarity in behavioural signatures (e.g., deficits in flexibility), the DSM-5 classifies PG (gambling disorder) as an addictive disorder that is separate from OCD and impulse-control disorders [27,30,33,34]. Our findings are consistent with the possibility that compulsive behaviours observed in the two disorders are driven by a similar computational process (i.e., asymmetric reward learning) but implemented by distinct neural mechanisms (the dmPFC and caudate in OCD and the insula in PG).

### Caveats and limitations of the study

One caveat of this study is its real-world significance. There was no significant difference in basic behaviour (i.e., the overall task performance and reaction time) between the OCD/PG and HC groups. Furthermore, we found no significant correlations between decision parameters (i.e., learning rate and perseveration) and questionnaire-based measurements of symptom severity within each group. Moreover, it is worth noting that there was no direct evidence of behavioural inflexibility in our sample of OCD and PG patients. We did not collect explicit measures of behavioural inflexibility, and generalised linear regression did not reveal any significant differences in the effect of past choices on behaviour between the OCD/PG and HC groups. The relationship between asymmetric learning rates obtained from our RL modelling and behavioural inflexibility observed in the real world (e.g., excessive hand washing and pathological gambling) remains elusive. Taken together, future studies should be conducted to evaluate the real-world significance of this task.

A limitation of this study is the small sample size, which may result in low statistical power. Our study’s sample size (34 HC, 29 OCD patients, and 17 PG patients) was comparable to those of other neuroimaging studies that recruited patients with OCD/PG [37–39,50,73,80,83]. However, it was smaller than the sample size recommended for exploring individual differences [85]. Therefore, caution must be exercised when interpreting and applying this study’s findings because low statistical power could increase the likelihood of obtaining false-negative and false-positive results [86].

In this study, the two clinical groups were recruited from different channels: OCD from clinical services and PG from the local community (see Methods). Furthermore, the proportion of the participants with the intake of SSRIs was higher in OCD than in PG (S1 Table, Fisher’s exact test; OCD versus PG: *P* = 0.023). These factors could lead to the differential behavioural and neural signatures of RL [87–89].

## Conclusions

Grounded in a well-characterised RL framework, the present study elucidates neurocomputational mechanisms of reward-learning anomalies in patients with OCD and PG. We demonstrate that OCD and PG were associated with altered learning rates from positive and negative reward prediction errors. Our findings are consistent with the possibility that asymmetric sensitivity to better- and worse-than-expected outcomes in reward-learning regulates, at least in part, abnormal compulsive behaviours in these disorders. More broadly, the present study sheds light on the importance of a computational model that bridges behaviour and neural activity in a unified framework (e.g., RL), facilitating more comprehensive explanations of the pathophysiology of mental disorders.

## Methods

This study was approved by the Human Research Ethics Committee of Monash University (ID: 1238896).

### Participants

We recruited 34 participants diagnosed with OCD, 23 with PG, and 39 HC as part of a broader project [32,90,91]. No statistical methods were used to predetermine the sample size; however, our sample size was consistent with those used in previous studies [37,39,50,73,83]. All participants provided informed written consent.

Participants with OCD were recruited from specialist clinical services located in Melbourne, VIC, Australia. Participants with PG and HCs were recruited from the local community. The participants were pre-assessed to exclude those with a lifetime history of concussion, neurological disease, or drug abuse/dependence.

All the participants underwent phone screening, followed by an in-depth face-to-face assessment. The first screening over the phone included the short MINI International neuropsychiatric interview screener, the Florida Obsessive-Compulsive Inventory [92], and the Problem Gambling Severity Index [54]. In the second screening, LP and LB performed the full MINI International neuropsychiatric interview for OCD and other mental disorders and the structured clinical interview for PG in DSM-IV to further characterise the participants’ symptoms. Participants with OCD were screened to ensure that their severity section score on the Florida obsessive-compulsive inventory was >8, and their diagnosis was confirmed using treatment services and the MINI International neuropsychiatric interview. Participants with PG who engaged in electronic gambling at least once a week were screened to ensure that their problem gambling severity index score was >8, and their diagnosis was confirmed by the Structured Clinical Interview for DSM-IV. Participants with OCD or PG who had either depression or anxiety (as indexed by the MINI) were not excluded as long as the OCD and PG symptoms constituted the primary cause of distress and interference in the participants’ lives. Participants were excluded if they met the criteria for any other psychiatric disorder, including the concurrent presence of OCD and PG.

Furthermore, on the day of the second screening, we obtained the Obsessive-Compulsive Inventory-Revised (OCI-R) [53], Barratt Impulsiveness Scale [93], Beck Depression Inventory [94], and State and Trait Anxiety Inventory [95] scores as indices of the severity of psychiatric symptoms.

### Exclusion criteria

For the analyses, we excluded data from participants who failed to respond to >20% of the trials in any of the four main conditions (reward and avoidance trials conducted in sessions 1 and 2). We also excluded those who chose only one option in more than one of the four conditions. The remaining data used for the subsequent analyses included 34 HC (18 females; age 34.6 ± 9.8 years; 33 right-handed), 29 OCD (16 females; age 32.3 ± 10.1; 29 right-handed), and 17 PG (7 females; age 33.6 ± 12.9; 17 right-handed) participants. See S1 Table for the demographic and clinical information.

### Stimuli

We used six pairs of 12 fractal images in the experiment. Each pair was presented to the participants in one of the three types of trials in each of the two sessions. The association between image pairs and trial types was randomised across participants.

### Decision-making task

Each participant performed a probabilistic instrumental learning task (Fig 1A). In this task, each of two sessions contained randomly interleaved 30 *reward*, 30 *avoidance*, and 30 *neutral* trials (i.e., one of the three trials was presented at pseudo-random on each trial). Participants chose between two stimuli to earn rewards in the reward trials and made choices to avoid losses in the avoidance trials. In the neutral trials, a visual image not associated with monetary incentives was presented. Note that different pairs of stimuli were presented in the three types of trial (see Fig 1A).

In the reward/avoidance trials, one of the paired stimuli yielded a reward/loss with a probability of approximately 0.7, whereas the other yielded a reward/loss with a probability of approximately 0.3. In the neutral trials, the two stimuli led to a neutral feedback with probabilities of approximately 0.7 and 0.3, respectively. In each session, the stimulus with a higher probability of reward/loss was randomly reversed once between the 10th and 20th trial without any explicit cue (the timing was independent across the three types of trials). The reversal was introduced to foster participants’ learning over the course of all trials. Note that the reversal often favours model-based learning algorithms with an adaptive rate [96], but such models did not outperform the original models in this study (S1 Text). Furthermore, unlike a conventional reversal-learning task in which one of the two options was rewarding in each trial, in our task, the probabilistic outcomes of the two stimuli were determined independently. The participants did not receive any instructions regarding the structure of the reward/loss environment.

In each trial, participants chose between the two fractal images by pressing a button with their right hand within 2 s (decision phase; Fig 1A). The two images were positioned randomly to the left or right of the screen in each trial. After the response, the chosen image was highlighted by an increase in brightness (anticipation phase, 3.5 s). The outcome of the choice was then revealed by visual feedback (outcome phase, 1.5 s; Fig 1A): a picture of a store card with the text “You win 1 point!” indicated a reward; a red cross on top of the card with the text “You lose 1 point!” indicated a loss; a scrambled picture of the card with the text “Nothing” indicated a neutral outcome; and a blank screen with a fixation cross indicated no reward and no loss. Note that the neutral outcome was not associated with any reward or loss and had essentially the same meaning as the blank screen in terms of monetary incentives.

### Reward payment

The participants were informed that they would receive a gift card corresponding to the number of points earned in the tasks. However, all the participants received a $20 gift card that could be used in an Australian major department store (Myer) and a supermarket (Coles). The deception was employed in the experimental design due to ethical concerns regarding paying PG patients based on their performance. However, it is worth noting that we replicated well-known striatal and prefrontal responses to the reward prediction error and the expected value signals (S7 Fig) [62,63], supporting the validity of our experiment to examine reward-based decision-making.

### Behavioural data analysis

#### GLMM1

To examine the effects of the past outcome and choice on behaviour, we constructed a generalised linear mixed-effects model for each of the 3 trial types and the 3 groups (i.e., 9 models in total, called GLMM1 collectively): logit *P* (choice = *A*) ~ 1 + *R*_*t*-1_ + *C*_*t*-1_ + (1 | participant), where *R*_*t*-1_ and *C*_*t*-1_ denote the outcome and choice in the previous trial (i.e., the previous occasion where the same trial type was presented) [36]. *R*_*t*-1_ was coded as 1 if the participant chose option *A* and obtained a reward (loss) in the previous trial, -1 if they chose option *B* and obtained a reward (loss), and 0 if there was no reward (loss). *C*_*t*-1_ was coded as 1 if the participant chose option *A* in the previous trial and -1 otherwise. The term (. | participants) indicates that the variables were considered as random effects (i.e., were allowed to vary between participants). We also tested models that included the random effects of *R*_*t*-1_ and *C*_*t*-1_; however, the complicated models were not favoured by the Akaike information criterion (AIC).

#### GLMM2

We performed additional GLMM analyses to test for group differences (HC versus OCD and HC versus PG) in the effects of the past outcome and choice: logit *P* (choice at *t* = *A*) ~ 1 + *R*_*t*-1_ + *C*_*t*-1_ + *d*_*OCD*_ x *R*_*t*-1_ + *d*_*OCD*_ x *C*_*t*-1_ + *d*_*PG*_ x *R*_*t*-1_ + *d*_*PG*_ x *C*_*t*-1_ + *d*_*OCD*_ + *d*_*PG*_ + (1 | participant), where *d*_*OCD*_ and *d*_*PG*_ are dummy variables indicating the OCD and PG groups. Here, the interaction terms denote how the effects of the past reward and choice were modulated by mental disorders (compared with HC).

#### Correction for multiple comparisons

To control for the type I error rate in the behavioural analyses, we employed Bonferroni correction for the number of tests performed (see each of the figure legends for details).

### Computational models

We considered four RL models. RL1 was a conventional one, in which the value of the chosen option was updated in proportion to the reward prediction error with learning rate *α*. RL2 had different learning rates, *α*_(+)_ and *α*_(-)_, for positive and negative reward prediction errors, respectively. RL3 included a perseveration parameter, *γ*, denoting the tendency to repeat the same choice. RL4 included both differential learning rates and perseveration.

#### RL1

In this simple Q-learning model [9], an individual’s decision was guided by the values of the two available options, *Q*(*A*) and *Q*(*B*). These values governed the participant’s probability of choosing option *A* as follows: *q*(*A*) = 1 / [1 + exp (-*β* (*Q*(*A*)–*Q*(*B*)))], where the parameter *β*>0 (*inverse temperature*) controls for the degree of noise in the decision-making. Once the outcome of the choice is revealed, the value of the chosen option is updated by the reward prediction error. That is, when option *A* is chosen, the value is updated as follows: *Q*(*A*)←*Q*(*A*)+*α δ*. Here, *δ* is the reward prediction error, defined as *δ* = *R*–*Q*(*A*), where *R* denotes the reward outcome (1 for a reward, -1 for a loss, and 0 for no-reward or no-loss). The parameter *α*∈[0,1] is called the *learning rate* and controls the speed of learning with respect to the reward prediction error.

#### RL2

This model included differential learning rates for positive and negative reward prediction errors [97,98]. Specifically, the value of the chosen option, say *A*, was updated as follows:

$$Q(A)\leftarrow \left\{ \begin{array}{l} Q(A)+\alpha_{\left(+\right)}\delta \;\mathrm{if}\;\delta \geq 0\\ Q(A)+\alpha_{\left(-\right)}\delta \;\mathrm{if}\;\delta <0 \end{array}\right.,$$

where *α*_(+)_ and *α*_(-)_ indicate the asymmetric learning rates.

#### RL3

The third model included the perseveration effect, in which the constant bonus *γ* was added to the option chosen in the previous trial (i.e., the previous occasion where the same trial type was presented) [37,38]. Specifically, the probability of choosing option *A* was given by *q*(*A*) = 1 / [1 + exp (-*β* (*Q*(*A*)–*Q*(*B*) + *γ*)] if *A* had been chosen in the previous trial, and by 1 / [1 + exp (-*β* (*Q*(*A*)–*Q*(*B*))–*γ*)] when *B* had been chosen. The parameter *γ* denotes the degree of perseveration (i.e., tendency to repeat the same choice). The process of value updating (i.e., learning) was the same as that in RL1.

#### RL4

The fourth model included differential learning rates and perseveration term.

These models were fitted to the participants’ choice data in each group (HC, OCD, and PG) and each trial type (reward and avoidance trials). For this, we used a hierarchical modelling approach to reduce the estimation noise in the parameter estimates [55] (see S3A Fig for the details of RL3). Each participant’s individual-level parameters were assumed to be drawn from common group-level normal distributions: e.g., for participant *i*, the parameters were defined as logit(*α*^(*i*)^) ~ *N*(*μ*_*α*_, *σ*_*α*_), log(*β* ^(*i*)^) ~ *N*(*μ*_*β*_, *σ*_*β*_), and*γ* ^(*i*)^ ~ *N*(*μ*_*γ*_, *σ*_*γ*_). The posterior distributions of the group-level parameters were estimated using the variational Bayes framework in *CmdStanR* version 2.26.1, as sampling methods (e.g., Markov chain Monte Carlo) were not practically feasible for exhaustive model- and parameter-recovery analyses (see *Validation of the model fitting based on the simulation data* below). Weekly informative priors were set for the group-level parameters: *N*(0,3) for *μ*_*α*_ and *μ*_*γ*_,, and *N*(0,1) for *μ*_*β*_, *σ*_*α*_,, *σ*_*β*_,, and *σ*_*γ*_. We confirmed that the main results of the model fitting (Figs 3 and 4) did not change by ensuring flatter priors for the group-level parameters: *N*(0,5) for *μ*_*α*_ and *μ*_*γ*_, and *N*(0,2) for *μ*_*β*_, *σ*_*α*_,, *σ*_*β*_,, and *σ*_*γ*_; or *N*(0,9) for *μ*_*α*_ and *μ*_*γ*_, and *N*(0,3) for *μ*_*β*_, *σ*_*α*_,, *σ*_*β*_,, and *σ*_*γ*_.

Each model’s goodness of fit was assessed using the WAIC [56], obtained from the posterior distributions using the *R* package “*loo*” (version 2.4.1). The WAIC is an advanced version of AIC, which is applicable to a wide range of cases (e.g., hierarchical modelling).

To examine group differences in the estimated parameters, we focused on the posterior distributions of the mean differences (Fig 4B, 4C, 4E, and 4F). We calculated the Bayes Factor (BF: the strength of evidence against the null hypothesis that the group difference is *zero*) using the Savage–Dickey method [99]. Conventionally, BF >150 is judged as “very strong,” 20 to 150 as “strong,” and 3 to 20 as “positive evidence” [100].

### Validation of the model fitting based on the simulation data

We ran the simulation 300 times. In each simulation run, the data of 30 agents were generated by each of the three computational models (i.e., RL1, RL2, and RL3). To cover the ranges of the parameters to a reasonable degree, we assumed that *α*, *α*_(+)_, and *α*_(-)_ were sampled from *Beta*(1, 1), that *β* was from *N*(3.5, 0.5), and that *γ* was sampled from *N*(0.4, 0.2).

#### Parameter recovery analysis

In the simulation data, we first examined whether the generative parameter values could be recovered by hierarchical Bayesian model fitting [57]. For each parameter in each model, we first plotted the fitted values against the true generative values (S3B–S3D Fig, *top*), and then quantified the recoverability by the correlation between the two values (S3B–S3D Fig, *bottom*; histograms of the correlation coefficients over 300 simulation runs).

#### Model recovery analysis

We next verified the identifiability of the models by constructing a “confusion matrix” and an “inversion matrix” [57] (S3E Fig). In the confusion matrix, each row denoted the probability of each of the three competing models providing the best fit (i.e., with the lowest WAIC value) for the data generated by the corresponding model. In the inversion matrix, each row denoted the probability that the data best fitted by the corresponding model (i.e., with the lowest WAIC value) was generated from each of the three competing models.

#### Replication of the results of model-neutral analysis

The data simulated by the best-fitted models and their parameter estimates successfully replicated the main behavioural findings obtained in the model-neutral analysis (S5 Fig).

### fMRI data acquisition

We collected fMRI images using a 3T Siemens MAGNETOM Skyra Syngo MR D13C scanner at Monash Biomedical Imaging (Clayton, VIC, Australia). The BOLD signal was measured using a one-shot T2*-weighted echo planar imaging sequence (volume TR = 2,000 ms, TE = 30 ms, FA = 90°). We acquired 34 oblique slices (thickness, 3.0 mm; gap, 0 mm; FOV, 230 × 230 mm; matrix, 76 × 76) per volume. After the two functional runs (479 volumes each), high-resolution (1 mm^3^) anatomical images were acquired using a standard MPRAGE pulse sequence (TR = 2,300 ms, TE = 2.07 ms, FA = 9°). The data were analysed using the SPM12 software in MATLAB R2020b on a MacBook Pro (16-inch, 2019; Mac OS X 10.17.7). Data from one OCD participant were excluded due to technical problems with the fMRI scan.

### fMRI data analysis

We analysed the fMRI data to identify the neural correlates of model-derived reward prediction errors in the reward and avoidance trials (compared to the counterparts in the neutral trials). The comparison with the data from neutral trials was important to control for confounding effects, such as visual responses to the outcome feedback [101]. The counterparts of the reward prediction error in the reward and avoidance trials were the original and inverted prediction errors in the neutral trials, respectively. Note that in the reward trials, reward prediction error was heightened with the unexpected presentation of a visual feedback of reward and lowered with the unexpected omission of the visual feedback. In the avoidance trials, reward prediction error was heightened with the unexpected omission of a visual feedback of loss and lowered with the unexpected presentation of the visual feedback. In the neutral trials, the prediction error was heightened with the unexpected presentation of a visual feedback of the neutral outcome and lowered with the unexpected omission of the visual feedback. Given the nature of the prediction error in each trial type, we compared the neural encoding of prediction error in the reward trials to that of the prediction error in the neutral trials, and the neural encoding of prediction error in the avoidance trials to that of the inverse prediction error in the neutral trials.

#### Preprocessing

We employed a standard procedure in SPM12: After slice timing correction, the images were realigned to the first volume to correct for the participants’ movements, spatially normalised based on the segmentation of the anatomical image, and spatially smoothed using an 8 mm FWHM Gaussian kernel. High-pass temporal filtering (filter width = 128 s) was also applied to the data.

#### GLM1

The GLM contained parametric regressors representing the unitary reward prediction errors at the time of the outcome (Figs 1A and 5). Specifically, the participant-specific design matrices contained the following regressors: two boxcar functions for the decision (duration = response time) and outcome (duration = 1.5 s) phases, and one stick function at the time of the key response (duration = 0 s). Missed trials were modelled as separate regressors. We included three parametric modulators of the boxcar function for the outcome phase that encoded the reward prediction errors in the reward, avoidance, and neutral trials. To control for the nuisance effects of decision-related neural signals, we included three modulators in the decision phase that encoded the value of the chosen option in the reward, avoidance, and neutral trials. All regressors were convolved with a canonical hemodynamic response function (with the serial orthogonalization of parametric modulators turned off). In addition, six motion-correction parameters were included as regressors of no interest to account for motion-related artefacts.

#### GLM2

This GLM contained separate parametric regressors representing the positive and negative parts of the reward prediction error (Figs 1A and 5). That is, the design matrices contained six parametric modulators of the outcome phase: (1) the positive prediction error in the reward trials; (2) the negative prediction error in the reward trials; (3) the positive prediction error in the avoidance trials; (4) the negative prediction error in the avoidance trials; (5) the positive prediction error in the neutral trials; and (6) the negative prediction error in the neutral trials. The other settings were the same as those used for GLM1.

#### Whole-brain analysis

As described in the main text, we used a two-step approach. First, we identified the brain regions tracking the reward prediction error independent of the mental disorder diagnosis by averaging the data of all the participants. We obtained the weighted average using the inverse of the number of participants in each group as weights, which indicated the ROIs that were statistically independent of the subsequent across-group comparisons. To this end, we defined contrasts of interest for the prediction errors weighted by the inverse of the number of participants in each group (HC: *N* = 34, OCD: *N* = 29, and PG: *N* = 17) as follows: 17/34 for HC, 17/29 for OCD, and 1 for PG following normalisation by the number of PG patients. For each participant, the contrasts were estimated at every voxel of the whole brain and entered into a random-effects analysis. We set our significance threshold at *P* < 0.05, which was whole-brain corrected for multiple comparisons at the cluster level (based on the conservative cluster-forming threshold at *P* < 0.0005, uncorrected [102]).

#### ROI analysis

Second, we tested whether the neural representations differed between the OCD/PG and HC groups. ROI analyses were performed using the MarsBaR toolbox for SPM. Each ROI was defined as a sphere (radius: 9 mm = 3 voxels) centred on the peak coordinates in a cluster of interest (S3–S5 Tables). Group differences in the effect sizes of the neural representations (i.e., regression coefficients in the fMRI GLM) were examined using the two-tailed Welch’s *t* test with Bonferroni correction for the number of tests performed (see each of the figure legends for details).

## Supporting information

S1 Text Supplementary note 1 (model fitting). (PDF)

S1 Fig Questionnaire-based psychiatric symptoms. (PDF)

S2 Fig Supplementary regression analysis on the behaviour. (PDF)

S3 Fig Supplementary behavioural analysis on the simulated data. (PDF)

S4 Fig Supplementary results of the model fitting with RL4. (PDF)

S5 Fig Supplementary behavioural analysis on the simulated data. (PDF)

S6 Fig Supplementary analysis of the parameter estimation. (PDF)

S7 Fig Replication of the previous neuroimaging findings in HC. (PDF)

S8 Fig Neuroimaging analysis for the left and right insula. (PDF)

S9 Fig Comparisons between SSRI-medicated and unmedicated OCD patients. (PDF)

S10 Fig Comparisons between the neuroimaging results with and without z-normalisation of reward prediction errors. (PDF)

S1 Table Demographic characteristics of the participants. (PDF)

S2 Table Correlations between symptom severity and RL parameters. (PDF)

S3 Table Brain areas exhibiting significant changes in the BOLD signal associated with the positive reward prediction error in reward trials. (PDF)

S4 Table Brain areas exhibiting significant changes in the BOLD signal associated with the negative reward prediction error in reward trials. (PDF)

S5 Table Brain areas exhibiting significant changes in the BOLD signal associated with the unitary reward prediction error in avoidance trials. (PDF)

S6 Table WAIC values in the supplementary model comparison. (PDF)

S7 Table WAIC values in the supplementary model comparison. (PDF)

## Abbreviations

ACC: anterior cingulate cortex
AIC: Akaike information criterion
BF: Bayes factor
fMRI: functional magnetic resonance imaging
GLMM: generalised linear mixed model
HC: healthy control
HDI: highest density interval
mPFC: medial prefrontal cortex
OCD: obsessive-compulsive disorder
OCI-R: obsessive-compulsive inventory-revised
PG: pathological gambling
RL: reinforcement learning
ROI: regions of interest
SSRI: selective serotonin reuptake inhibitor
WAIC: widely applicable Akaike information criterion
