# Supplementary material for: Individuals with problem gambling and obsessive-compulsive disorder learn through distinct reinforcement mechanisms
Source: PLoS Biol. 2023 Mar 14;21(3):e3002031. doi: 10.1371/journal.pbio.3002031 (PMC10013903; doi:10.1371/journal.pbio.3002031)
Supplement: S1 Text — (PDF) [file pbio.3002031.s001.pdf]

S1 Text. Supplementary note 1 (model fitting)

In the RESULTS section of the main text, we found that, in the reward trials, the PG group exhibited a higher learning rate for positive reward prediction errors and a lower rate for negative prediction errors than the HC (Fig 4ac). Moreover, the OCD group was found to have a lower learning rate for negative prediction errors than the HC (Fig 4ab).

Here, we describe that (i) RL3 and RL4 are not well-dissociable, and that (ii) the key findings above (i.e., group differences in the learning rates) did not change whether RL4 was included or not. First, model recovery analysis on the simulated data showed that the 'confusion' and 'inversion' matrices [1] were not close to the identity matrix in the case with the *four* models (RL1, RL2, RL3, and RL4) (S4a Fig). For instance, if the true generative model was RL4, the best-fit model was identified as RL3 with the probability 0.37 and RL4 with the probability 0.47, demonstrating poor identifiability between the two models. Second, comparing the four models, we found that RL4 provided the best fit for the HC group (S4b Fig); but the key findings (i.e., group differences of the learning rates in the reward trials) remained untouched (S4cd Fig).

Next, we tested for the Volatility Kalman Filter (VKF) and Pearce-Hall (PH) models [2,3]. These models modulate the learning rate depending on the estimated volatility of the environment and, therefore, could exploit the structure of the decision-making task: i.e., the reward/loss probability for each option drifted, and the option with a higher probability was reversed without any explicit cue. We also tested for a RELATIVE model [4,5]. This model adapts the range of outcomes in each context (i.e., reward-seeking and loss-avoidance decision-making). However, model comparison showed that these additional models did not outperform the best-fitted RL models in either the reward or the avoidance trial (S6 Table).

Finally, we compared parsimonious models, which share a common set of parameters across the two trial types, with the best-fitted models in the original analysis (RL2 in the reward trials and RL3 in the avoidance trials). We also compared an RL model with motor-perseveration (i.e., the tendency to choose options presented on the same side in consecutive trials), in which a constant bonus is added to the option presented on the same side as that chosen in the last trial irrespective of the trial types. These models did not provide better predictions (S7 Table).

1. Wilson RC, Collins AG. Ten simple rules for the computational modeling of behavioral data. Elife. 2019;8: e49547. doi:10.7554/elife.49547

2. Piray P, Daw ND. A simple model for learning in volatile environments. Plos Comput Biol. 2020;16: e1007963. doi:10.1371/journal.pcbi.1007963

3. Li J, Schiller D, Schoenbaum G, Phelps EA, Daw ND. Differential roles of human striatum and amygdala in associative learning. Nat Neurosci. 2011;14: 1250–1252. doi:10.1038/nn.2904

4. Burke CJ, Baddeley M, Tobler PN, Schultz W. Partial Adaptation of Obtained and Observed Value Signals Preserves Information about Gains and Losses. J Neurosci. 2016;36: 10016–10025. doi:10.1523/jneurosci.0487-16.2016

5. Palminteri S, Khamassi M, Joffily M, Coricelli G. Contextual modulation of value signals in reward and punishment learning. Nat Commun. 2015;6: 8096. doi:10.1038/ncomms9096
