# Supplementary material for: Individuals with problem gambling and obsessive-compulsive disorder learn through distinct reinforcement mechanisms
Source: PLoS Biol. 2023 Mar 14;21(3):e3002031. doi: 10.1371/journal.pbio.3002031 (PMC10013903; doi:10.1371/journal.pbio.3002031)
Supplement: S1 Fig — (PDF) [file pbio.3002031.s002.pdf]

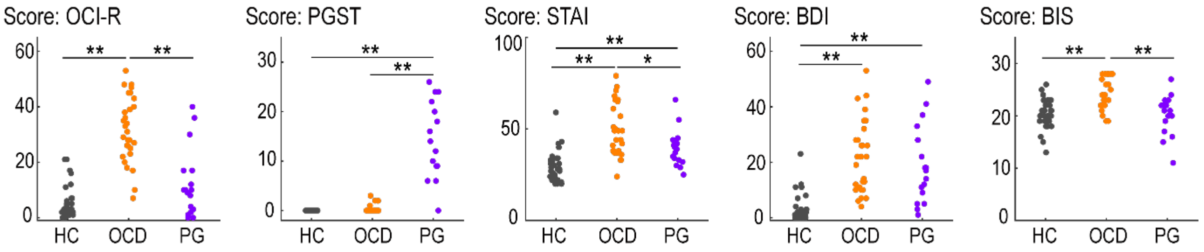

S1 Fig. Questionnaire-based psychiatric symptoms.

Obsessive-Compulsive Inventory-Revised (OCI-R), Problem Gambling Severity Index (PGSI), Barratt Impulsiveness Scale (BIS), Beck Depression Inventory (BDI), and the State and Trait Anxiety Inventory (STAI). **$P < 0.01$ and *$P < 0.05$, two-tailed Welch's *t*-test, Bonferroni-corrected for the three tests performed.

Summary data to reproduce the figure are available at https://osf.io/v7em5/.
