# Supplementary material for: Individuals with problem gambling and obsessive-compulsive disorder learn through distinct reinforcement mechanisms
Source: PLoS Biol. 2023 Mar 14;21(3):e3002031. doi: 10.1371/journal.pbio.3002031 (PMC10013903; doi:10.1371/journal.pbio.3002031)
Supplement: S2 Fig — (PDF) [file pbio.3002031.s003.pdf]

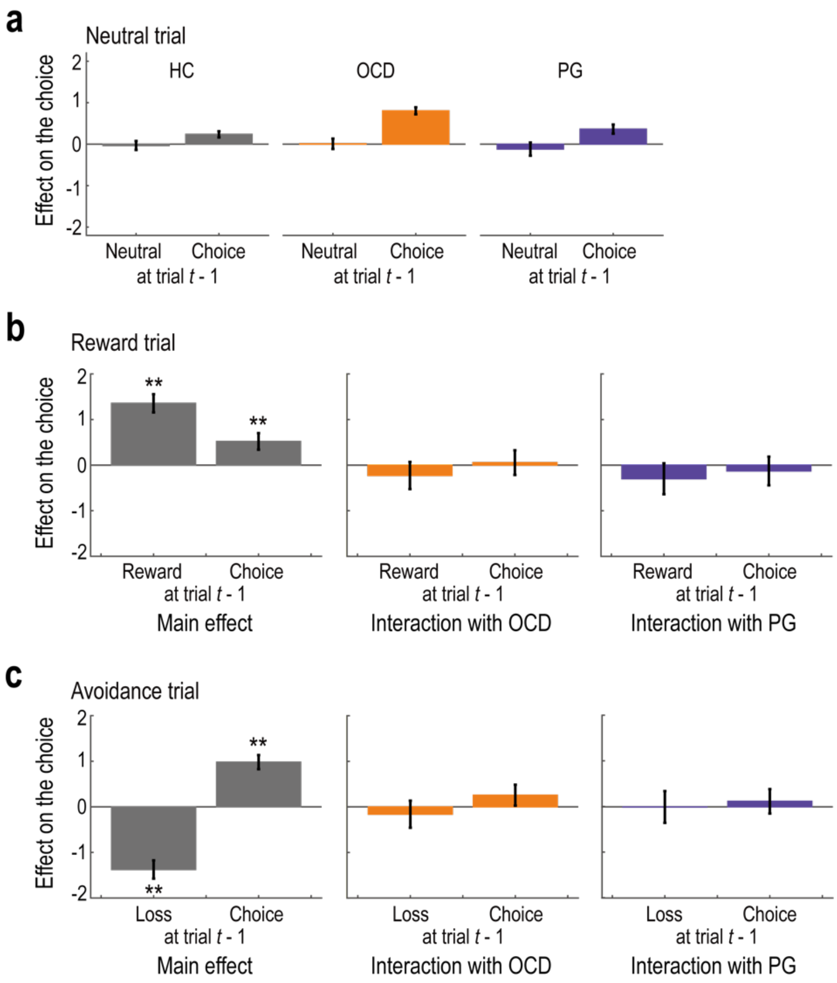

S2 Fig. Supplementary regression analysis on the behaviour.

(a) Effects of past neutral outcomes and past choices on current behaviour in neutral trials (mean ± SEM). The format is the same as in Fig 2.

(b) Effects of past rewards and past choices on current behaviour in reward trials (mean ± SEM). The mean and SEM of the effects were estimated with generalized linear mixed-effect models (GLMM2). **$P < 0.01$, two-tailed t-test, Bonferroni-corrected for the two tests performed in each model. *Grey*, the main effects over the three groups of participants (HC, OCD and PG). *Orange*, the interaction effects with the OCD dummy variable, indicating how the effects of the past reward and choice were modulated by OCD (compared with HC). *Purple*, the interaction effects with the PG dummy variable, indicating how the effects of the past reward and choice were modulated by PG (compared with HC).

(c) Effects of past losses and past choices on current behaviour in avoidance trials (mean ± SEM). The format is the same as in (b).

Summary data to reproduce the figure are available at https://osf.io/v7em5/.
