# Supplementary material for: Individuals with problem gambling and obsessive-compulsive disorder learn through distinct reinforcement mechanisms
Source: PLoS Biol. 2023 Mar 14;21(3):e3002031. doi: 10.1371/journal.pbio.3002031 (PMC10013903; doi:10.1371/journal.pbio.3002031)
Supplement: S3 Fig — (PDF) [file pbio.3002031.s004.pdf]

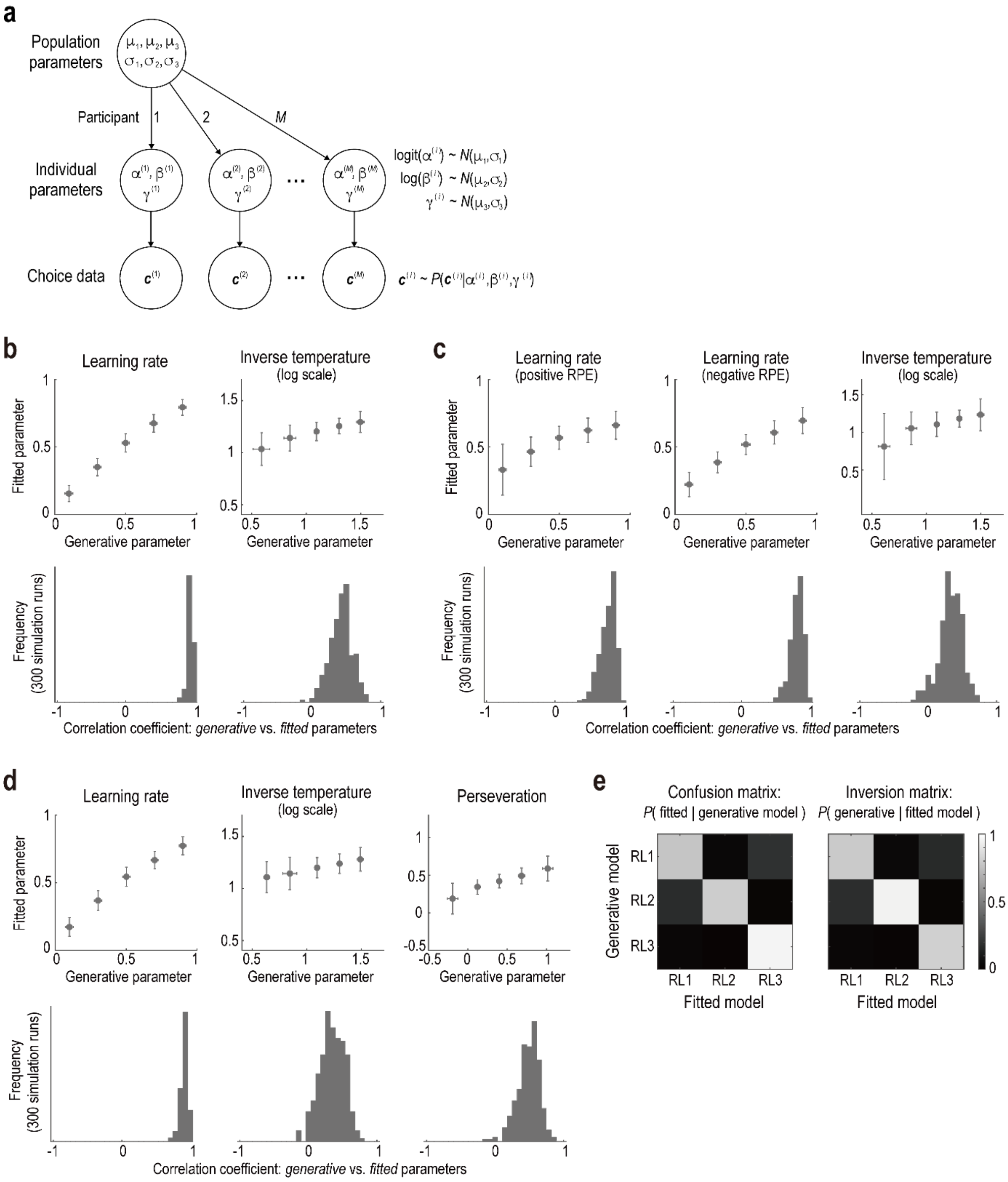

S3 Fig. Supplementary behavioural analysis on the simulated data.

(a) Graphical illustration of the hierarchical approach to model fitting. For each group (HC, OCD and PG), participant *i* 's individual-level parameters (e.g., $\alpha^{(i)}$, $\beta^{(i)}$ and $\gamma^{(i)}$ in RL3) are drawn from the common population-level normal distributions. *M*, the number of participants. $\boldsymbol{c}^{(i)}$, participant *i* 's choice data.

(b) Parameter recovery analysis for RL1. We recovered the parameter values by fitting the model to the simulated data (see Methods). *Top*, plots of the fitted parameters against the true generative ones in the simulation (Mean $\pm$ SD across 300 simulation runs).

Bottom, histograms of the correlation between the fitted and the true generative parameters over the 300 simulation runs.

(c) Parameter recovery analysis for RL2. The format is the same as in (b).

(d) Parameter recovery analysis for RL3. The format is the same as in (b).

(e) Model recovery analysis. *Left*, confusion matrix: each row denotes the probability that each of the three competing models provides the best fit on the data generated by the corresponding model. *Right*, inversion matrix: each row denotes the probability that the data best fitted by one model is actually generated from each of the three competing models.

Summary data to reproduce the figure are available at https://osf.io/v7em5/.
