# Supplementary material for: Individuals with problem gambling and obsessive-compulsive disorder learn through distinct reinforcement mechanisms
Source: PLoS Biol. 2023 Mar 14;21(3):e3002031. doi: 10.1371/journal.pbio.3002031 (PMC10013903; doi:10.1371/journal.pbio.3002031)
Supplement: S4 Fig — (PDF) [file pbio.3002031.s005.pdf]

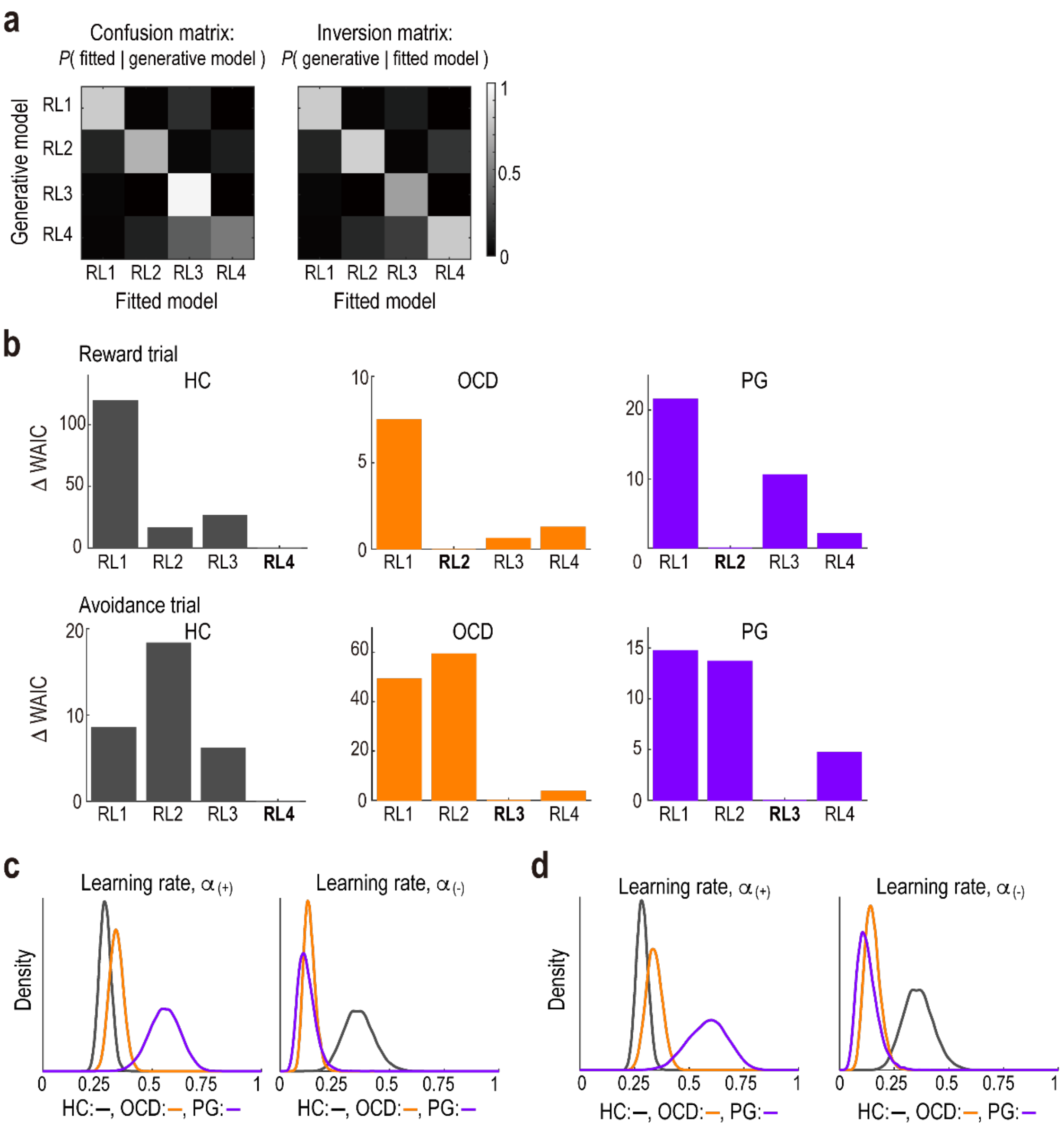

S4 Fig. Supplementary results of the model fitting with RL4.

(a) Model recovery analysis on the simulated data. *Left*, confusion matrix: each row denotes the probability that each of the four competing models (RL1, RL2, RL3, and RL4) provides the best fit on the data generated by the corresponding model. *Right*, inversion matrix: each row denotes the probability that the data best fitted by one model is actually generated from each of the four competing models.

(b) Model comparison. We plotted each model's WAIC value relative to the best model (note: a smaller value indicates a better fit). The best-fitted model is highlighted in bold. WAIC, widely applicable Akaike information criterion; RL, reinforcement learning; HC, healthy control; OCD, obsessive-compulsive disorder; PG, pathological gambling.

(c) Learning rates in the best-fitted models in reward trials. The parameter estimates were obtained from RL4 in the HC group and from RL2 in the OCD and the PG groups. Posterior distributions of group-level mean parameters were plotted. *Left*, learning rate

from the positive reward prediction error, $\alpha_{(+)}$. *Right*, learning rate from the negative reward prediction error, $\alpha_{(-)}$. *Grey*, healthy control (HC); *orange*, obsessive-compulsive disorder (OCD); *purple*, pathological gambling (PG).

(d) Learning rates in RL4 in reward trials. The format is the same as in (c). The parameter estimates were obtained using RL4 in all HC, OCD, and PG groups.

Summary data to reproduce the figure are available at https://osf.io/v7em5/.
