# Supplementary material for: Individuals with problem gambling and obsessive-compulsive disorder learn through distinct reinforcement mechanisms
Source: PLoS Biol. 2023 Mar 14;21(3):e3002031. doi: 10.1371/journal.pbio.3002031 (PMC10013903; doi:10.1371/journal.pbio.3002031)
Supplement: S5 Fig — (PDF) [file pbio.3002031.s006.pdf]

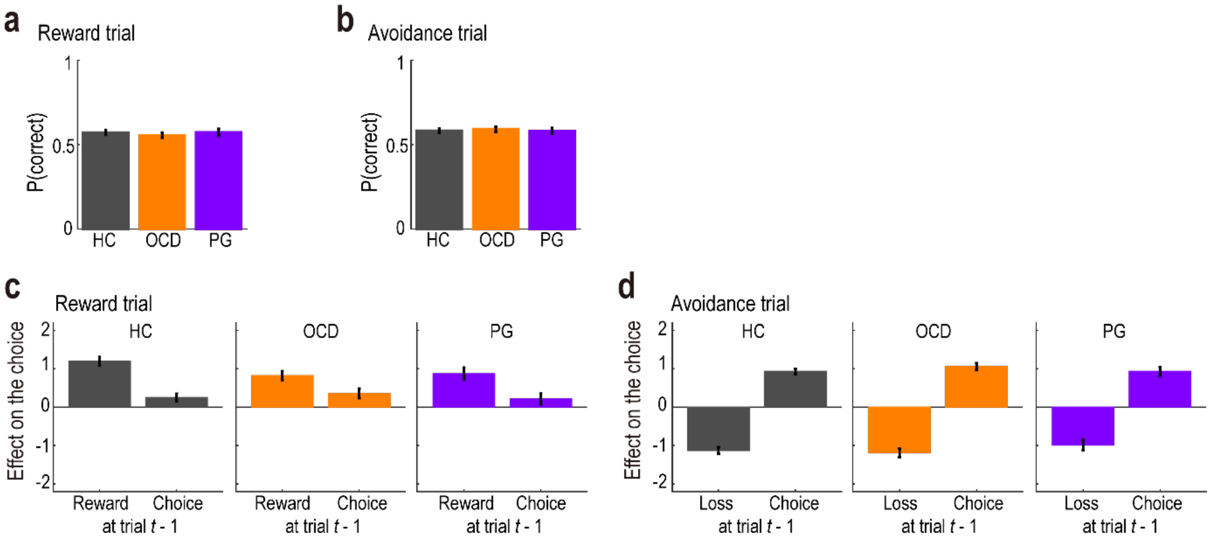

S5 Fig. Supplementary behavioural analysis on the simulated data.

(a) Proportion of correct choices in reward trials (mean ± SD). The correct choice was defined as selecting the option that provided the higher reward (or lower loss) probability in the given trial. HC, healthy control; OCD, obsessive-compulsive disorder; and PG, pathological gambling.

(b) Proportion of correct choices in avoidance trials (mean ± SD). The format is the same as in (a).

(c) Effects of past rewards and choices on current behaviour in reward trials (mean ± SEM). The format is the same as in Fig 2a.

(d) Effects of past losses and choices on current behaviour in avoidance trials. The format is the same as in Fig 2b.

Summary data to reproduce the figure are available at https://osf.io/v7em5/.
