# Supplementary material for: Individuals with problem gambling and obsessive-compulsive disorder learn through distinct reinforcement mechanisms
Source: PLoS Biol. 2023 Mar 14;21(3):e3002031. doi: 10.1371/journal.pbio.3002031 (PMC10013903; doi:10.1371/journal.pbio.3002031)
Supplement: S6 Fig — (PDF) [file pbio.3002031.s007.pdf]

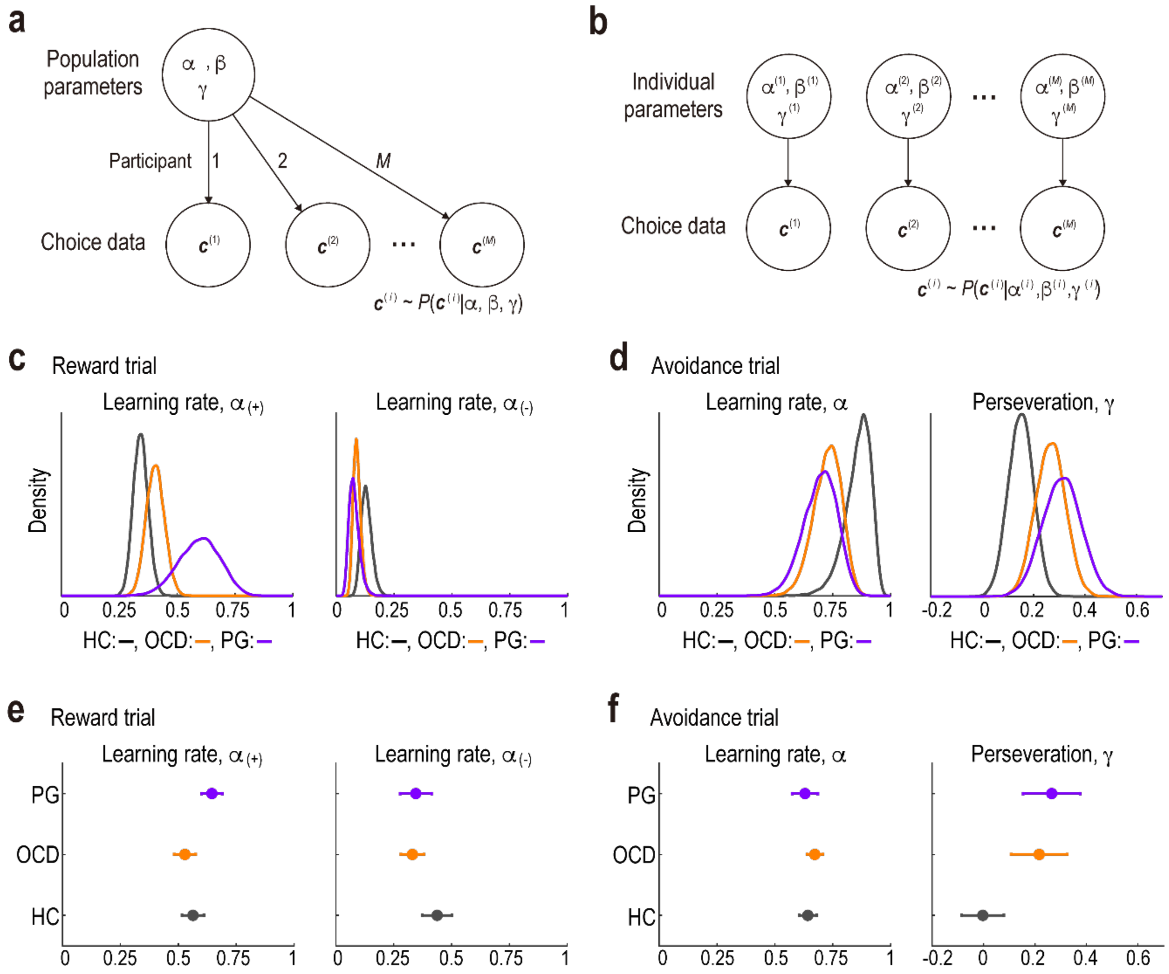

S6 Fig. Supplementary analysis of the parameter estimation.

(a) Graphical illustration of the *group* model-fitting approach that pools the data from all participants together. For each group (HC, OCD and PG), participants are assumed to share the same set of parameters (e.g., α, β and γ in RL3). *M*, the number of participants. $\boldsymbol{c}^{(i)}$, participant *i* 's choice data.

(b) Graphical illustration of the *individual* model-fitting approach that assumes each participant has an independent set of parameters (e.g., $\alpha^{(i)}$, $\beta^{(i)}$ and $\gamma^{(i)}$ in RL3). The model is fitted to each participant's data separately.

(c) Results of the *group* model-fitting approach in reward trials: posterior distributions of parameters of RL2 for each group. *Left*, learning rate from the positive reward prediction error, $\alpha_{(+)}$ (HC: mode = 0.34 and 95% HDI = [0.28, 0.41]; OCD: mode = 0.41 and 95% HDI = [0.33, 0.48]; and PG: mode = 0.62 and 95% HDI = [0.42, 0.76]). *Right*, learning rate from the negative reward prediction error, $\alpha_{(-)}$ (HC: mode = 0.13 and 95% HDI = [0.09, 0.17]; OCD: mode = 0.09 and 95% HDI = [0.06, 0.12]; and PG: mode = 0.07 and 95% HDI = [0.04, 0.12]). *Grey*, healthy control (HC); *orange*, obsessive-compulsive disorder (OCD); *purple*, pathological gambling (PG).

(d) Results of the *group* model-fitting approach in avoidance trials: posterior distributions of

parameters of RL3 for each group. *Left*: learning rate, α (HC: mode = 0.88 and 95% HDI = [0.75, 0.95]; OCD: mode = 0.75 and 95% HDI = [0.61, 0.84]; and PG: mode = 0.72 and 95% HDI = [0.55, 0.83]); *right*: perseveration, γ (HC: mode = 0.16 and 95% HDI = [0.06, 0.24]; OCD: mode = 0.28 and 95% HDI = [0.15, 0.38]; and PG: mode = 0.32 and 95% HDI = [0.16, 0.45]). The format is the same as in (c).

(e) Results of the *individual* model-fitting approach in reward trials: MEAN and SEM of the individual participants' parameter estimates. *Left*, learning rate from the positive reward prediction error, $\alpha_{(+)}$ (HC: MEAN $\pm$ SEM = 0.56 $\pm$ 0.05; OCD: 0.53 $\pm$ 0.05; and PG: 0.65 $\pm$ 0.04). *Right*, learning rate from the negative reward prediction error, $\alpha_{(-)}$ (HC: 0.44 $\pm$ 0.06; OCD: 0.33 $\pm$ 0.05; and PG: 0.35 $\pm$ 0.07). *Grey*, healthy control (HC); *orange*, obsessive-compulsive disorder (OCD); *purple*, pathological gambling (PG).

(f) Results of the *individual* model-fitting approach in avoidance trials: MEAN and SEM of the individual participants' parameter estimates. *Left*: learning rate, α (HC: 0.64 $\pm$ 0.04; OCD: 0.67 $\pm$ 0.03; and PG: 0.63 $\pm$ 0.06); *right*: perseveration, γ (HC: 0.00 $\pm$ 0.08; OCD: 0.22 $\pm$ 0.11; and PG: 0.26 $\pm$ 0.11). The format is the same as in (e).

Summary data to reproduce the figure are available at https://osf.io/v7em5/.
