# Supplementary material for: Individuals with problem gambling and obsessive-compulsive disorder learn through distinct reinforcement mechanisms
Source: PLoS Biol. 2023 Mar 14;21(3):e3002031. doi: 10.1371/journal.pbio.3002031 (PMC10013903; doi:10.1371/journal.pbio.3002031)
Supplement: S7 Fig — (PDF) [file pbio.3002031.s008.pdf]

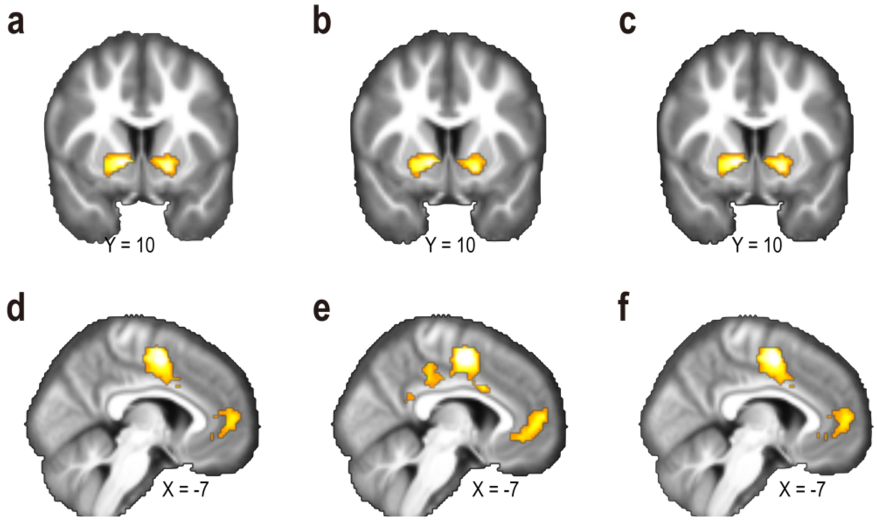

S7 Fig. Replication of the previous neuroimaging findings in HC.

(a) Neural correlates of the unitary reward prediction error signal in HC. Activity in the ventral striatum and other regions were significantly correlated with the reward prediction error at the time of outcome delivery ($P < 0.05$ corrected at cluster-level). The reward prediction error signal was estimated using RL1. The map is thresholded at $P < 0.0005$ uncorrected for display purpose.

(b) Neural correlates of the unitary reward prediction error signal in HC. The reward prediction error signal was estimated using RL2. The format is the same as in (a).

(c) Neural correlates of the unitary reward prediction error signal in HC. The reward prediction error signal was estimated using RL3. The format is the same as in (a).

(d) Neural correlates of value signal in HC. Activity in the medial prefrontal cortex was significantly correlated with the value of the chosen option at the time of decision-making ($P < 0.05$ corrected at cluster-level). The value signal was estimated using RL1. The format is the same as in (a).

(e) Neural correlates of the value signal in HC. The value signal was estimated using RL2. The format is the same as in (a).

(f) Neural correlates of the value signal in HC. The value signal was estimated using RL3. The format is the same as in (a).

Summary data to reproduce the figure are available at https://osf.io/v7em5/.
