# Supplementary material for: Individuals with problem gambling and obsessive-compulsive disorder learn through distinct reinforcement mechanisms
Source: PLoS Biol. 2023 Mar 14;21(3):e3002031. doi: 10.1371/journal.pbio.3002031 (PMC10013903; doi:10.1371/journal.pbio.3002031)
Supplement: S8 Fig — (PDF) [file pbio.3002031.s009.pdf]

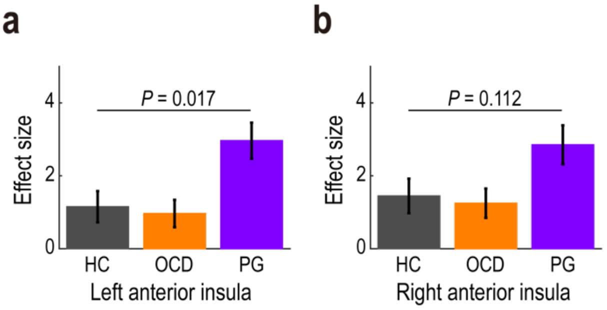

S8 Fig. Neuroimaging analysis for the left and right insula.

(a) Activity in the left insula at the time of outcome correlated with the positive reward prediction error in reward trials. The *P* value in a two-tailed Welch's *t*-test is Bonferroni-corrected for the two tests performed. The format is the same as in Fig 6a.

(b) Activity in the right insula at the time of outcome correlated with the positive reward prediction error in reward trials. The format is the same as in Fig 6a.

Summary data to reproduce the figure are available at https://osf.io/v7em5/.
