# Supplementary material for: Individuals with problem gambling and obsessive-compulsive disorder learn through distinct reinforcement mechanisms
Source: PLoS Biol. 2023 Mar 14;21(3):e3002031. doi: 10.1371/journal.pbio.3002031 (PMC10013903; doi:10.1371/journal.pbio.3002031)
Supplement: S9 Fig — (PDF) [file pbio.3002031.s010.pdf]

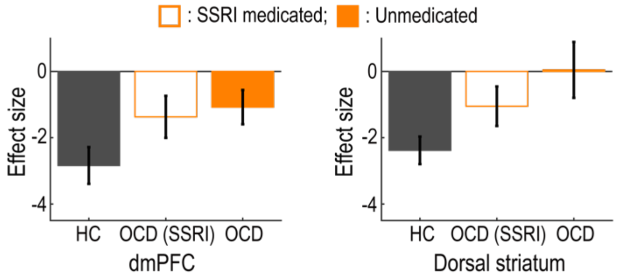

S9 Fig. Comparisons between SSRI-medicated and unmedicated OCD patients.
Effect sizes of the negative reward prediction error in reward trials for the HC, SSRI-medicated OCD, and unmedicated OCD groups (mean ± SEM). *Left*, dorsomedial prefrontal cortex (dmPFC); and *right*, dorsal striatum. *Grey bars*, HC; *open bars*, SSRI-medicated OCD; and *orange bars*, unmedicated OCD. HC, healthy control; OCD, obsessive-compulsive disorder; SSRI, selective serotonin reuptake inhibitors.
Summary data to reproduce the figure are available at https://osf.io/v7em5/.
