# Supplementary material for: Individuals with problem gambling and obsessive-compulsive disorder learn through distinct reinforcement mechanisms
Source: PLoS Biol. 2023 Mar 14;21(3):e3002031. doi: 10.1371/journal.pbio.3002031 (PMC10013903; doi:10.1371/journal.pbio.3002031)
Supplement: S10 Fig — (PDF) [file pbio.3002031.s011.pdf]

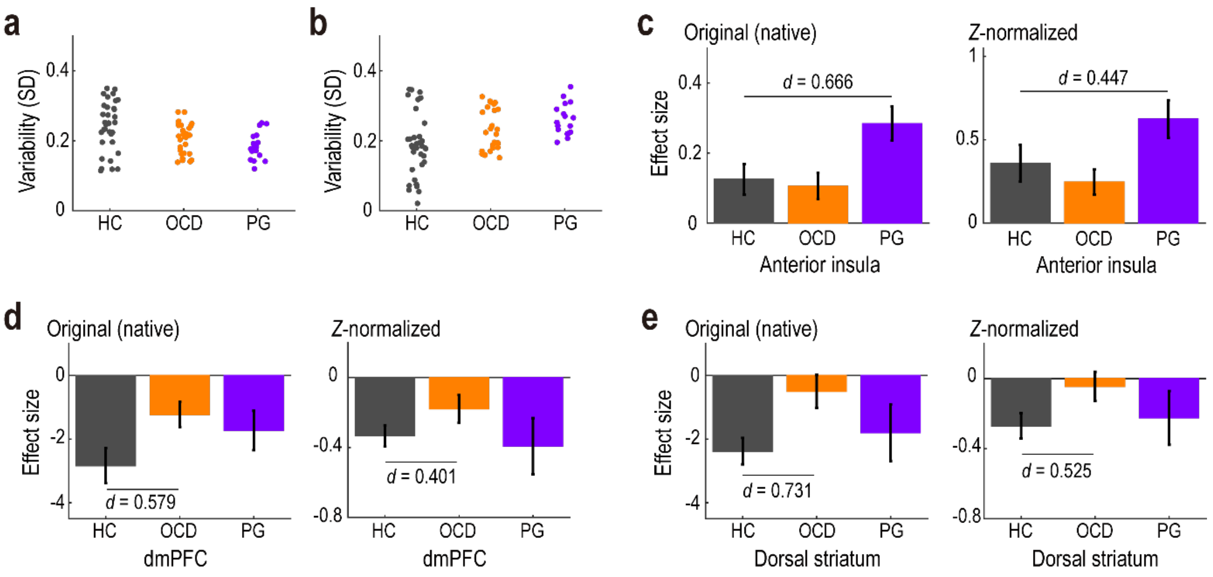

S10 Fig. Comparisons between the neuroimaging results with and without z-normalisation of reward prediction errors.

(a) Variability of positive reward prediction error in reward trials. Each point denotes the standard deviation of the prediction error in one participant. SD, standard deviation.

(b) Variability of negative reward prediction error in reward trials. The format is the same as in (a).

(c) Positive reward prediction error in the anterior insula for reward trials: PG vs HC (left: *Cohen's d* = 0.666, *t* = 2.430, and *P* = 0.020; right: *Cohen's d* = 0.447, *t* = 1.683, and *P* = 0.100). The format is the same as in Fig. 6a. *Left*, the original fMRI data analysis results without z-normalisation of the prediction error. *Right*, results of the post-hoc fMRI data analysis with z-normalisation of the prediction error. *d* indicates the effect size, *Cohen's d*.

(d) Negative reward prediction error in the dmPFC for reward trials: OCD vs HC (left: *Cohen's d* = 0.579, *t* = 2.359, and *P* = 0.022; right: *Cohen's d* = 0.401, *t* = 1.543, and *P* = 0.129). The format is the same as in (c).

(e) Negative reward prediction error in the dorsal striatum for reward trials: OCD vs HC (left: *Cohen's d* = 0.731, *t* = 2.831, and *P* = 0.006; right: *Cohen's d* = 0.525, *t* = 2.050, and *P* = 0.045). The format is the same as in (c).

Summary data to reproduce the figure are available at https://osf.io/v7em5/.
