# Supplementary material for: Individuals with problem gambling and obsessive-compulsive disorder learn through distinct reinforcement mechanisms
Source: PLoS Biol. 2023 Mar 14;21(3):e3002031. doi: 10.1371/journal.pbio.3002031 (PMC10013903; doi:10.1371/journal.pbio.3002031)
Supplement: S1 Table — (PDF) [file pbio.3002031.s012.pdf]

S1 Table. Demographic characteristics of the participants

	HC (*N* = 34)	OCD (*N* = 29)	PG (*N* = 17)
Age (mean ± SD)	34.6 ± 9.8 yrs	32.3 ± 10.1 yrs	33.6 ± 12.9 yrs
Sex (% females)	56%	55%	41%
% Right-handers	97%	100%	100%
IQ (mean ± SD)	114.6 ± 13.1	115.9 ± 10.0	113.1 ± 12.3
Illness duration (mean ± SD)	N/A	13.9 ± 8.99 yrs	9.43 ± 6.72 yrs
Monthly monetary losses from gambling (mean ± SD)	6.8 ± 22.5 AU$	10.1 ± 27.4 AU$	1318.8 ± 1735.0 AU$
SSRI Medication (%)	0%	48%	12%

HC, healthy control; OCD, obsessive-compulsive disorder; PG, pathological gambling; IQ, intelligence quotient (missing in one HC and one OCD participant); and SSRI, selective serotonin reuptake inhibitor.
