# Supplementary material for: Individuals with problem gambling and obsessive-compulsive disorder learn through distinct reinforcement mechanisms
Source: PLoS Biol. 2023 Mar 14;21(3):e3002031. doi: 10.1371/journal.pbio.3002031 (PMC10013903; doi:10.1371/journal.pbio.3002031)
Supplement: S2 Table — (PDF) [file pbio.3002031.s013.pdf]

S2 Table. Correlations between symptom severity and RL parameters.

		HC	OCD	PG
Reward trial	α_+ vs. OCI-R:	r = 0.153 (P = 0.389)	r = 0.059 (P = 0.760)	r = -0.036 (P = 0.891)
	α_+ vs. PGSI:	r = N/A (P = N/A)	r = -0.105 (P = 0.588)	r = -0.220 (P = 0.400)
	α_- vs. OCI-R:	r = -0.177 (P = 0.318)	r = -0.178 (P = 0.356)	r = -0.184 (P = 0.480)
	α_- vs. PGSI:	r = N/A (P = N/A)	r = 0.020 (P = 0.920)	r = -0.286 (P = 0.266)
Avoidance trial	α vs. OCI-R:	r = -0.133 (P = 0.452)	r = 0.163 (P = 0.397)	r = 0.115 (P = 0.659)
	α vs. PGSI:	r = N/A (P = N/A)	r = -0.104 (P = 0.593)	r = -0.216 (P = 0.404)
	γ vs. OCI-R:	r = -0.305 (P = 0.080)	r = 0.021 (P = 0.916)	r = -0.066 (P = 0.802)
	γ vs. PGSI:	r = N/A (P = N/A)	r = -0.084 (P = 0.663)	r = -0.081 (P = 0.758)

RL parameters: α_+, learning rate for the positive reward prediction error; α_-, learning rate for the negative reward prediction error; α, learning rate; and γ, perseveration. Symptom severity: OCI-R, total score of Obsessive-Compulsive Inventory-Revised; and PGSI, total score of Problem Gambling Severity Index. Note that in HC all the participants had *zero* score in PGSI and so the correlations with the RL parameters are not defined.
