# Supplementary material for: Individuals with problem gambling and obsessive-compulsive disorder learn through distinct reinforcement mechanisms
Source: PLoS Biol. 2023 Mar 14;21(3):e3002031. doi: 10.1371/journal.pbio.3002031 (PMC10013903; doi:10.1371/journal.pbio.3002031)
Supplement: S3 Table — (PDF) [file pbio.3002031.s014.pdf]

S3 Table. Brain areas exhibiting significant changes in the BOLD signal associated with the positive reward prediction error in reward trials.

Correlation	Region	Hemi	x	y	z	t-statistic	*p*-value	Voxels
Positive	**Insula**	R	30	20	-13	7.02	0.000	1618
	**Ventral striatum** (ventral part of the caudate and putamen)	R	12	8	2	6.80	0.000	-
	**Insula**	L	-30	20	-10	6.30	0.000	-
	**mPFC** (BA 8/32)	R	3	41	29	6.86	0.000	749
	**mPFC** (BA 32)		6	41	11	5.57		
	**mPFC** (BA 10)		0	62	17	4.92		
	Inferior parietal lobule (BA 7/40)	L	-48	-61	44	6.06	0.000	385
	Inferior parietal lobule (BA 7/40)	R	33	-79	11	5.87	0.000	569
	Middle occipital gyrus (BA 19)	L	-30	-88	20	5.84	0.000	69
	Middle temporal gyrus (BA 21)	R	66	-31	-4	5.02	0.000	92
	Posterior cingulate cortex (BA 23)	L/R	0	-28	35	4.94	0.000	153
	Lateral prefrontal cortex (BA 8/9)	R	42	20	53	4.78	0.000	80
	Precentral gyrus (BA 6/9)	L	-51	2	47	4.71	0.000	110
	Precuneus (BA 7)	R	9	-67	41	4.40	0.000	60
Negative	Paracentral lobule (BA 5)	R	9	-37	56	4.36	0.000	82

Activated clusters observed in the whole-brain analysis ($P < 0.05$ cluster-level corrected) of fMRI. The regions of interest discussed in the main text are shown in bold. mPFC: medial prefrontal cortex; BA, Brodmann area.
