# Supplementary material for: Individuals with problem gambling and obsessive-compulsive disorder learn through distinct reinforcement mechanisms
Source: PLoS Biol. 2023 Mar 14;21(3):e3002031. doi: 10.1371/journal.pbio.3002031 (PMC10013903; doi:10.1371/journal.pbio.3002031)
Supplement: S4 Table — (PDF) [file pbio.3002031.s015.pdf]

S4 Table. Brain areas exhibiting significant changes in the BOLD signal associated with the negative reward prediction error in reward trials.

Correlation	Region	Hemi	x	y	z	t-statistic	*p*-value	Voxels
Positive	Posterior cingulate cortex (BA 31)	L	-12	-55	17	6.91	0.000	937
	**vmPFC** (BA 32/10)	L	-3	47	-4	6.82	0.000	784
	**vmPFC** (BA 32)	L/R	0	35	-10	6.25	0.000	-
	**vmPFC** (BA 32)	L	-9	29	-4	6.00	0.000	-
	Middle occipital gyrus (BA 19/39)	L	-36	-76	38	5.68	0.000	89
	Hippocampus	R	21	-10	-16	5.43	0.000	75
	Hippocampus	L	-24	-22	-19	5.41	0.000	86
	Lateral prefrontal cortex (BA 8)	L	-21	38	50	4.78	0.000	52
	Lateral orbitofrontal cortex (BA 47)	L	-33	35	-13	4.78	0.000	35
	Operculum	R	36	-16	23	4.51	0.000	48
Negative	**dmPFC** (BA 8)	R	3	23	47	6.58	0.000	319
	**dmPFC** (BA 8)	R	3	35	44	6.32	0.000	-
	Lateral prefrontal cortex (BA 9/8/6)	R	45	14	41	6.39	0.000	1339
	**Insula**	R	36	20	-1	6.34	0.000	-
	Lateral prefrontal cortex (BA 9)	R	51	26	32	6.31	0.000	-
	Inferior parietal lobule (BA 40)	R	51	-40	53	6.21	0.000	538
	**Insula**	L	-30	20	2	5.21	0.000	144
	Inferior parietal lobule (BA 40)	L	-33	-55	50	5.00	0.000	239
	Lateral prefrontal cortex (BA 9/8/6)	L	-48	20	41	4.80	0.000	165
	**Striatum (caudate)**	R	12	5	11	4.62	0.000	79

Activated clusters observed in the whole-brain analysis ($P < 0.05$ cluster-level corrected) of fMRI. The regions of interest discussed in the main text are shown in bold. vmPFC: ventromedial prefrontal cortex; dmPFC: dorsomedial prefrontal cortex; BA, Brodmann area.
