# Supplementary material for: Individuals with problem gambling and obsessive-compulsive disorder learn through distinct reinforcement mechanisms
Source: PLoS Biol. 2023 Mar 14;21(3):e3002031. doi: 10.1371/journal.pbio.3002031 (PMC10013903; doi:10.1371/journal.pbio.3002031)
Supplement: S5 Table — (PDF) [file pbio.3002031.s016.pdf]

S5 Table. Brain areas exhibiting significant changes in the BOLD signal associated with the unitary reward prediction error in avoidance trials.

Correlation	Region	Hemi	x	y	z	t-statistic	*p*-value	Voxels
Positive	Lateral prefrontal cortex (BA 8/9)	R	21	56	8	6.40	0.000	1938
	Lateral prefrontal cortex (BA 46/10)	L	-45	44	8	5.89	0.000	218
	Inferior parietal lobule (BA 40/7)	L	-33	-55	41	5.73	0.000	1068
	Inferior parietal lobule (BA 7/40)	R	33	-55	41	5.51	0.000	1062
	Middle temporal gyrus (BA 37)	L	-42	-49	-10	5.05	0.000	68
	Inferior temporal gyrus (BA 37)	R	33	-67	2	4.98	0.000	176
	**Striatum (putamen)**	R	30	-13	-1	4.56	0.000	75
	**Striatum (putamen)**	L	-27	-10	2	4.25	0.000	52
Negative	**mPFC** (BA 10/9)	L	-6	53	23	4.98	0.000	150
	**mPFC** (BA 10)	L/R	0	56	17	4.73	0.000	-
	**mPFC** (BA 10)	L	-6	50	11	4.20	0.000	-

Activated clusters observed in the whole-brain analysis ($P < 0.05$ cluster-level corrected) of fMRI. The regions of interest discussed in the main text are shown in bold. mPFC: medial prefrontal cortex; BA, Brodmann area.
