# Supplementary material for: Individuals with problem gambling and obsessive-compulsive disorder learn through distinct reinforcement mechanisms
Source: PLoS Biol. 2023 Mar 14;21(3):e3002031. doi: 10.1371/journal.pbio.3002031 (PMC10013903; doi:10.1371/journal.pbio.3002031)
Supplement: S6 Table — (PDF) [file pbio.3002031.s017.pdf]

S6 Table. WAIC values in the supplementary model comparison.

	Group	Best-fitted RL	VKF	PH	RELATIVE
Reward trial	HC	**1814.4**	2265.4	1880.9	1915.0
	OCD	**1474.9**	1733.4	1487.0	1485.0
	PG	**999.2**	1214.3	1018.6	1011.8
Avoidance trial	HC	**2373.0**	2382.9	2380.9	2385.6
	OCD	**1929.6**	1969.6	1964.2	1985.7
	PG	**1165.5**	1170.2	1170.1	1177.4

WAIC, widely applicable Akaike information criterion; HC, healthy control; OCD, obsessive-compulsive disorder; PG, pathological gambling; Best-fitted RL, RL2 in reward trials and RL3 in avoidance trials; VKF, Volatility Kalman Filter model; PH, Pearce-Hall model; and RELATIVE, RELATIVE model.
