# Supplementary material for: Individuals with problem gambling and obsessive-compulsive disorder learn through distinct reinforcement mechanisms
Source: PLoS Biol. 2023 Mar 14;21(3):e3002031. doi: 10.1371/journal.pbio.3002031 (PMC10013903; doi:10.1371/journal.pbio.3002031)
Supplement: S7 Table — (PDF) [file pbio.3002031.s018.pdf]

S7 Table. WAIC values in the supplementary model comparison.

Group	Best-fitted RL	$RL1_{common}$	$RL2_{common}$	$RL3_{common}$	$RL_{motor\text{-}perseveration}$
HC	**4178.4**	4396.9	4275.5	4277.6	4369.3
OCD	**3387.4**	3642.0	3496.2	3509.0	3656.0
PG	**2160.1**	2250.5	2190.1	2171.5	2226.2

WAIC, widely applicable Akaike information criterion; HC, healthy control; OCD, obsessive-compulsive disorder; PG, pathological gambling; Best-fitted RL, RL2 in reward trials and RL3 in avoidance trials; $RL1_{common}$, RL1 that has a common set of parameters across the reward and the avoidance trials; $RL2_{common}$, RL2 that has a common set of parameters across the reward and the avoidance trials; $RL3_{common}$, RL3 that has a common set of parameters across the reward and the avoidance trials. $RL_{motor\text{-}perseveration}$, RL with motor-perseveration.
